# Chimpanzee pant‐hoots encode individual information more reliably than group differences

**DOI:** 10.1002/ajp.23430

**Published:** 2022-09-12

**Authors:** Nisarg P. Desai, Pawel Fedurek, Katie E. Slocombe, Michael L. Wilson

**Affiliations:** ^1^ Department of Anthropology University of Minnesota Minneapolis Minnesota USA; ^2^ Division of Psychology, Faculty of Natural Sciences University of Stirling Stirling UK; ^3^ Department of Psychology University of York York UK; ^4^ Department of Ecology, Evolution, and Behavior University of Minnesota St. Paul Minnesota USA; ^5^ Institute on the Environment University of Minnesota St. Paul Minnesota USA

**Keywords:** chimpanzee, dialects, pant‐hoot, vocal learning

## Abstract

Vocal learning, the ability to modify the acoustic structure of vocalizations based on social experience, is a fundamental feature of speech in humans (*Homo sapiens*). While vocal learning is common in taxa such as songbirds and whales, the vocal learning capacities of nonhuman primates appear more limited. Intriguingly, evidence for vocal learning has been reported in chimpanzees (*Pan troglodytes*), for example, in the form of regional variation (“dialects”) in the “pant‐hoot” calls. This suggests that some capacity for vocal learning may be an ancient feature of the *Pan‐Homo* clade. Nonetheless, reported differences have been subtle, with intercommunity variation representing only a small portion of the total acoustic variation. To gain further insights into the extent of regional variation in chimpanzee vocalizations, we performed an analysis of pant‐hoots from chimpanzees in the neighboring Kasekela and Mitumba communities at Gombe National Park, Tanzania, and the geographically distant Kanyawara community at Kibale National Park, Uganda. We did not find any statistically significant differences between the neighboring communities at Gombe or among geographically distant communities. Furthermore, we found differences among individuals in all communities. Hence, the variation in chimpanzee pant‐hoots reflected individual differences, rather than group differences. Thus, we did not find evidence of dialects in this population, suggesting that extensive vocal learning emerged only after the lineages of *Homo* and *Pan* diverged.

## Highlights

- Previous studies have reported dialects in chimpanzee pant‐hoot calls.
- Vocal dialects may reflect vocal learning ability.
- We compared two neighboring and one geographically distant chimpanzee communities.
- The acoustic structure of pant‐hoots reflected individual but not group identity.

**Abbreviations**
DAG: directed acyclic graph
DFA: discriminant function analysis
GLMM: generalized linear mixed model
PCA: principal component analysis
PC1, PC2, etc.: principal component 1, principal component 2, etc.
pDFA: permuted discriminant function analysis

## 1 INTRODUCTION

Vocal learning underlies the human capacity for speech. The desire to understand the evolution of this capacity motivates much of the research into vocal learning in other animals (Fitch, 2010). Over time, the definition of vocal learning has evolved as researchers have identified several nuances in vocal learning ability across animals. Janik and Slater (2000) defined vocal production learning broadly, as “signals modified in form as a result of experience with those of other individuals.” Other researchers have focused on more specific aspects, such as the ability to modify and learn new vocalizations through imitation (Fitch, 2010). Regardless of the particular definition used, it is clear that vocal learning has evolved independently multiple times in animals (Vernes et al., 2021). For example, songbirds (Passeriformes) (Cunningham & Baker, 1983) and humpback whales (*Megaptera novaeangliae*) (Garland et al., 2011) learn elaborate songs. Parrots (Psittaciformes) can mimic human speech, and distinguish group members from drifters based on learned vocalizations (Bartlett & Slater, 1999; Hile & Striedter, 2000).

In comparison to birds and whales, the vocal learning capacities of nonhuman primates appear much more limited (Fischer & Hammerschmidt, 2020). Evidence for active learning of new vocalizations by nonhuman primates remains modest (Tyack, 2020). Recent studies indicate that orangutans (*Pongo* spp.) can acquire a voiceless vocalization (whistle) in captivity (Wich et al., 2009), produce novel voiced vocalizations in controlled settings (e.g., using a membranophone [Lameira & Shumaker, 2019]), and exhibit differences in alarm call variants at different population densities; population density being a measure for sociality (Lameira et al., 2022). Some nonhuman primates have been reported to engage in vocal learning through modifying the acoustic structure of vocalizations based on auditory feedback and imitation. Takahashi et al. (2015) found that in common marmosets (*Callithrix jacchus*), parental feedback influences the rate of vocal development (Takahashi et al., 2015). Marmosets (*Callithrix* spp.) exhibit dialects in the form of geographical variation in their vocalizations in the wild (de la Torre & Snowdon, 2009) as well as population specific acoustic structure across call types in captivity (Zürcher & Burkart, 2017). Sugiura (1998) reported that Japanese macaques (*Macaca fuscata*) match some of the acoustic features of recorded “coo” calls during a playback experiment. Fischer et al. (2020) reported vocal convergence in the grunts of male Guinea baboons (*Papio papio*) as individuals that interacted more frequently with one another exhibited greater resemblance than the grunts of males that interacted less frequently.

Much of the literature on vocal learning in animals focuses on dialects, defined as regional variation in vocal production (Janik & Slater, 1997; Nowicki & Searcy, 2014). Such regional variation in vocal production could arise due to genetic differences among geographically distant communities, but among geographically adjacent communities, learning would seem to be a more likely mechanism (Filatova et al., 2012). When such variation is learned, it may signal membership in the local population (as in songbirds [Cunningham & Baker, 1983]), or membership in a particular social group, as in orcas (*Orcinus orca*) (Filatova et al., 2012). Studies of social birds and mammals have found that learned signals of group membership can benefit individual signalers in two main ways: (i) by eliciting affiliative interactions from group members and mates and/or (ii) by advertising group membership to rivals during agonistic interactions, such as during territory defense. For example, in birds, group‐specific calls appear to (i) help maintain social bonds among group members, as in budgerigars (*Melopsittacus undulatus*) (Farabaugh et al., 1994; Hile & Striedter, 2000); (ii) facilitate territory defense by helping individuals identify flock members and focus aggression on foreign callers, as in black‐capped chickadees (*Parus atricadpillus*) (Nowicki, 1983). Researchers have inferred similar functions in social mammals. For example, several species of toothed whales (Odontocetes) appear to use vocal dialects to facilitate spatial group cohesion and maintain social relationships (Janik, 2014; Tyack & Sayigh, 1997). Spatial cohesion in group‐living species facilitates maintaining social bonds, finding mates, and defending territories (Janik & Slater, 1998).

Although vocal data from all great ape species can provide useful information for understanding the evolution of language (Lameira & Call, 2020), historically, researchers interested in the origins of human language have particularly focused on the vocal behavior of chimpanzees (*Pan troglodytes*), as they are one of the two living species most closely related species to humans (Fedurek & Slocombe, 2011). The other closest living relative of humans, bonobos (*Pan paniscus*), remain relatively understudied (Gruber & Clay, 2016; de Waal & Lanting, 1998). Several studies from the field (Arcadi, 1996; Crockford et al., 2004; Mitani et al., 1992) and captivity (Marshall et al., 1999) have found evidence for regional variation (dialects) in chimpanzee “pant‐hoot” calls, which has been proposed to result from vocal learning (Crockford et al., 2004; Marshall et al., 1999). Pant‐hoots of males that spend more time together are more similar, and the acoustic features of their calls converge when chorusing together (Mitani & Brandt, 1994), suggesting a possible mechanism for the convergence of acoustic properties within groups (Fedurek, Schel, et al., 2013; Mitani & Gros‐Louis, 1998). Call convergence has also been reported for chimpanzee rough‐grunt calls in captivity (Watson et al., 2015a) (but see [Fischer et al., 2015] and [Watson et al., 2015b]). Chimpanzees live in groups with fission‐fusion dynamics, in which individuals travel in subgroups (known as “parties”) of varying size, and they communicate over long distances using vocalizations, often in noisy environments (Aureli et al., 2008; Eckhardt et al., 2015; Goodall, 1986; Marler & Tenaza, 1977). Thus, vocal dialects potentially facilitate spatial group cohesion, and territorial defense during intergroup encounters.

Chimpanzee pant‐hoots are structurally complex loud calls with a relatively consistent temporal patterning (Fedurek et al., 2016; Marler & Hobbett, 1975). The typical pattern consists of a sequence of four kinds of sound elements over a duration range of 2−20 s. Each sequence of similar elements is called a phase and so the pant‐hoots typically have four phases (see Section 2 for details). Of the four phases, the climax phase is the loudest, and can be heard most clearly over long distances. However, pant‐hoots exhibit considerable acoustic variation within and among individuals (Fedurek et al., 2016; Kojima et al., 2003; Marler & Hobbett, 1975; Mitani et al., 1996). The variation is not only limited to frequency properties of elements such as fundamental frequency, peak frequency, and so forth, but also involves variation in the number and presence/absence of different elements and phases (Supporting Information: Figures S3 (a−m)) (*ibid.*). Chimpanzees use pant‐hoots in a variety of intracommunity and intercommunity contexts. In intracommunity contexts, chimpanzees use pant‐hoot calls to communicate with members of their own community over long distances (Goodall, 1986). Pant‐hoots may function to communicate the caller's location to allies and associates within their own community (Goodall, 1986; Mitani & Brandt, 1994; Mitani & Nishida, 1993). Further, pant‐hoots play a role in facilitating social bonds as affiliative partners chorus more together (Fedurek, Machanda, et al., 2013) and play a role in regulating grouping dynamics by attracting allies and potential mates to the caller's location (Fedurek et al., 2014; Mitani & Nishida, 1993; Wrangham, 1977). In intercommunity contexts, interactions often involve hearing—and sometimes responding to—pant‐hoots from callers that are hundreds of meters away, far out of view (Wilson et al., 2012). The long‐distance nature of pant‐hoots allows chimpanzees to use pant‐hoots to advertise territory ownership (Wilson et al., 2007), and to signal numerical strength to members of neighboring communities during agonistic intergroup encounters (Herbinger et al., 2009; Wilson et al., 2001, 2012). Individual callers might thus benefit from encoding community‐specific cues. Playback experiments have demonstrated that chimpanzees can distinguish stranger pant‐hoots from those of familiar individuals (Herbinger et al., 2009) and that they are sensitive to numerical strength during intergroup encounters, being more likely to respond to simulated intruders when they are in parties with more males (Wilson et al., 2001). Hence, community‐specific dialects could play a role in cooperative defense by signaling community membership. While genetic similarity could lead to community‐specific vocalizations, socially learned signals of group membership might be useful in cases where not all group members are close genetic kin.

Despite these reasons for thinking that vocal dialects would benefit chimpanzees, current evidence raises several questions about the extent to which chimpanzees have socially learned signals of group membership. In the first study of chimpanzee dialects, Mitani et al. (1992) reported differences between Gombe and Mahale pant‐hoots and suggested that they may be an outcome of vocal learning. However, the differences among the communities were subtle compared to differences observed in songbirds (Cunningham & Baker, 1983) or whales (Garland et al., 2011). Mitani et al. (1992) found geographical differences in the composition of the build‐up phase, and in frequency properties of the climax phase. Mitani and Brandt (1994) later found that in a principal components analysis (PCA) of acoustic structure, community membership accounted for only 0%−11% of the variance on the principal components, compared to within‐individual factors (48%−79% of the variance) and between individual factors (17%−52% of the variance). Mitani further reassessed his findings, pointing out that since Gombe and Mahale are far from one another (~160 km) and likely genetically isolated, the acoustic differences may not necessarily represent vocal learning, but instead could represent genetic differences and/or body size (Mitani et al., 1999). Additionally, other environmental factors like habitat acoustics and/or sound environment might be more important in explaining the variation in such geographically distant communities.

In addition to assessing whether pant‐hoots signal group membership, researchers have studied the acoustic structure of pant‐hoots produced in different contexts, such as traveling, feeding, group fusion, and arrival at food sources (Clark & Wrangham, 1993, 1994; Fedurek et al., 2016; Goodall, 1986; Mitani & Nishida, 1993; Notman & Rendall, 2005; Uhlenbroek, 1996; Wrangham, 1977). Some studies reported an association of some properties of the let‐down phase of the pant‐hoots with the context (Clark & Wrangham, 1993; Fedurek et al., 2016; Notman & Rendall, 2005). Notman and Rendall (2005) and Uhlenbroek (1996) reported an association of the tonal structure of the climax scream element of the pant‐hoots with the context of the production. While this variation may provide information about context to receivers, Notman and Rendall (2005) argued that these differences are unlikely to be an outcome of vocal learning and are more likely to reflect arousal states of chimpanzees when calling. In any case, the context of the call production is a covariate that may need to be controlled for when testing for group differences (refer to the methods and the directed acyclic graph in Figure 2). Finally, as several studies have noted previously, pant‐hoots are individually distinctive (Fedurek et al., 2016; Kojima et al., 2003; Marler & Hobbett, 1975; Mitani et al., 1996). Signaling individual identity, rather than group membership, might therefore be the primary function of these calls.

To test the extent to which the acoustic structure of pant‐hoots specifically signals community membership and arises out of vocal learning via auditory feedback, three questions need to be answered: (i) Do the calls contain features that reliably indicate community membership, allowing chimpanzees to distinguish extra‐community pant‐hoots based on those features alone, rather than through familiarity with the calls of particular individuals? (ii) Do chimpanzees from neighboring communities have more distinct pant‐hoots than those from geographically distant communities? Greater differences among neighboring communities compared to geographically distant communities would indicate that chimpanzees are actively modifying the acoustic structure of pant‐hoots to differentiate their calls from those of neighbors. (iii) Does community membership explain vocal similarity better than genetic relatedness? Crockford et al. (2004) addressed all three of these questions by comparing genotyped individuals in three neighboring communities and one more distant community in Taï National Park, Côte d'Ivoire. They found that neighboring communities differed from one another more than they differed from the distant community, despite neighboring communities inhabiting adjacent areas of similar continuous forest environment, which supports the view that chimpanzees learned to produce an acoustic structure distinct to their own community. These findings thus support the view that vocal learning accounts for the acoustic differences among communities. However, due to the small number of available males in the communities, this study could only include three individuals per group, resulting in a small sample size. This raises the possibility that the findings are a statistical artifact resulting from small sample size. While it is well known that small sample sizes may led to false negatives, it is also the case that small sample size with noisy data can artificially exaggerate effect sizes and lead to false positives (Loken & Gelman, 2017). Hence, more studies are needed to replicate these findings to have more confidence in the results.

As a step toward re‐evaluating the role of vocal learning in chimpanzee calls, we recorded pant‐hoot calls from two neighboring chimpanzee communities in Gombe National Park, Tanzania and the geographically distant Kanyawara community of chimpanzees in Kibale National Park, Uganda. The objective of this study is to assess the extent to which variation in the acoustic structure of the pant‐hoots can be explained by community membership. To that end, we test two hypotheses. Our first hypothesis is: the acoustic structure of pant‐hoots contains features that reliably indicate community membership. In line with Crockford et al. (2004), if vocal learning shapes the acoustic structure of pant hoots into community‐specific dialects, we would expect to find greater differences in the structure of calls in the two neighboring Gombe communities, compared to the geographically distant Kanyawara community. Our second hypothesis is: the acoustic structure of pant‐hoots contains cues of individual identity more than community identity. While these are not mutually exclusive hypotheses (i.e., one or both or neither could be supported), they provide a framework for our research questions.

## 2 METHODS

### 2.1 Subjects and study sites

We studied chimpanzees at two study sites: Gombe National Park, Tanzania and Kibale National Park, Uganda. In Gombe, we studied two neighboring communities: Kasekela and Mitumba. In Kibale, we studied the chimpanzees of the Kanyawara community. Gombe is located in western Tanzania, along the shore of Lake Tanganyika (4°40′S, 29°38′E). At the time of the study, Gombe had three contiguous communities of chimpanzees, two of which (Kasekela and Mitumba) were well habituated and were followed nearly every day, throughout the day, as part of the long‐term research at Gombe. Kibale is located in western Uganda (0°33′N, 30°21′E). We analyzed calls recorded as a part of a previous chimpanzee vocal communication study at Kanyawara (Fedurek, Schel, et al., 2013). Following initial observations by Isabirye‐Basuta in 1983−1985 (Isabirye‐Basuta, 1988), the Kanyawara chimpanzees have been studied continuously since 1987 (Emery Thompson et al., 2020; Wrangham et al., 1992).

For this study, we included male chimpanzees of ages ≥14 year. By age 14, chimpanzees in our study communities are socially and sexually mature, and critically, previous research has shown that relevant milestones for mature pant‐hoot production have been reached by this age. By age 14, male chimpanzees at Gombe exhibit a marked increase in their rate of pant‐hoot production, (Fig. 14, [Pusey, 1990]) and their body weight approximates that of older adult males (Fig. 8, [Pusey et al., 2005]).

During the study period at Gombe (July 2016 to December 2017), we recorded calls from *N* = 8 males of the Kasekela community and *N* = 5 males of the Mitumba community. We compared calls from Gombe to those of 7 males of the geographically distant Kanyawara chimpanzee community in Kibale recorded during October 2010 to September 2011.

### 2.2 Data collection

N. P. D. and M. L. W. trained two Tanzanian field assistants, Nasibu Zuberi Madumbi and Hashim Issa Salala, to conduct focal follows and record chimpanzee vocalizations at Gombe. They used a Sennheiser ME66 shotgun microphone with K6 power module and a Marantz PMD661 MKII audio recorder. They recorded the vocalizations with a 96 kHz sampling frequency and a 16‐bit amplitude resolution. They conducted focal follows of individual males with the goal of recording as many calls as possible from the focal male, throughout the day. In addition to recording calls from the focal target, they also opportunistically recorded as many other calls as possible from known individuals to obtain the maximum number of calls. For each recording, they noted additional information including caller behavior, context, location, and party composition. Here, the recordings were obtained when the caller was traveling, feeding (or arriving at a feeding site), displaying, and resting (not traveling, feeding, or displaying) contexts. If pant‐hoots provide any information about food, an individual could produce them when they see food and also when consuming food. Hence, a pant‐hoot given when arriving at a patch with visible food was considered feeding context. Furthermore, in situations where multiple contexts overlapped, we included the highest priority context based on the following hierarchy: travel > feed > display > rest. To ensure sufficient sample sizes and consistency with recordings from Kanyawara, we limited analysis for context differences, and those where context was relevant, to calls recorded in traveling and feeding contexts and only included individuals with at least three calls recorded in both contexts. While the field assistants recorded all call‐types from both males and females, here we focus on pant‐hoots from males, because (1) pant‐hoots have been the focus of previous dialect studies; (2) they can be heard from far away, making them plausible signals of community membership, and (3) males produce pant‐hoots more often than females (Wilson et al., 2007).

From July 2016 to December 2017 the team recorded a total of *N* = 1252 calls (*N* = 884 from Kasekela and *N* = 368 from Mitumba). We reviewed these recordings and found that *N* = 723 (*N* = 481 from Kasekela and *N* = 242 from Mitumba) were of sufficiently high quality for acoustic analyses. These recordings consisted of a variety of calls including pant‐hoots, pant‐grunts, rough‐grunts, waa‐barks, and screams. Of the pant‐hoots in these recordings, some were choruses (where multiple individuals pant‐hoot together), and not all were from identified individuals. Choruses that had overlapping elements from multiple callers were excluded, as such overlap makes it harder to extract meaningful acoustic features from known individual callers. Further, to optimize both the number of recordings per individual and the total number of individuals included in the analyses, we excluded individuals that had fewer than 8 pant‐hoot call recordings. Based on this criterion, we excluded two individuals from the Kasekela community: Ferdinand (FE) and Gimli (GIM). While high‐ranking males usually call most frequently (Wilson et al., 2007), the highest‐ranking male at the start of our study, FE, was overthrown in October 2016, after which we were unable to record any more pant‐hoots from him. In Mitumba, in July 2017, the alpha male Edgar (EDG) killed one of the adult males Fansi (FAN) (Massaro et al., 2021). Before this, we were able to record enough calls from FAN for some analyses. These selection criteria yielded a total of 214 pant‐hoots (*N* = 128 from Kasekela and *N* = 86 from Mitumba) from 11 individuals (*N* = 6 males from Kasekela and *N* = 5 males from Mitumba) for acoustic analysis (Table 1).

**Table 1:** Number of pant‐hoots by each individual in the two contiguous Kasekela and Mitumba communities at Gombe National Park, Tanzania and one geographically distant Kanyawara community at Kibale National Park, Uganda included in this study

National park	Community	Individual	Age at beginning (years)	Total pant‐hoots	Pant‐hoots with climax screams	Pant‐hoots with build‐ups	Pant‐hoots per context
Gombe	Kasekela (*N* = 128)	Fundi (FND)	16	11	11	6	Feed: 1 Travel: 3
		Faustino (FO)	26	20	19	12	Feed: 7 Travel: 5
		Fudge (FU)	19	33	33	27	Feed: 8 Travel: 6
		Sheldon (SL)	33	15	12	14	Feed: 1 Travel: 6
		Sampson (SN)	19	38	35	34	Feed: 22 Travel: 5
		Zeus (ZS)	22	11	8	7	Feed: 1 Travel: 9
	Mitumba (*N* = 86)	Edgar (EDG)	27	45	41	24	Feed: 27 Travel: 9
		Fansi (FAN)	14	8	8	3	Feed: 2 Travel: 1
		Kocha (KOC)	15	16	16	12	Feed: 4 Travel: 6
		Lamba (LAM)	14	9	9	5	Feed: 3 Travel: 3
		Londo (LON)	15	8	8	6	Feed: 4 Travel: 0
Kibale	Kanyawara (*N* = 111)	Big Brown (BB)	44	8	8	6	Feed: 7 Travel: 1
		Eslom (ES)	15	18	18	10	Feed: 9 Travel: 9
		Kakama (KK)	25	21	21	20	Feed: 9 Travel: 12
		Makokou (LK)	28	14	14	14	Feed: 7 Travel: 7
		Twig (PG)	22	10	10	6	Feed: 3 Travel: 7
		Stout (ST)	55	14	14	11	Feed: 7 Travel: 7
		Lanjo (TJ)	15	26	26	9	Feed: 23 Travel: 3

*Note*: Total pant‐hoots include pant‐hoots from resting, displaying, and unknown contexts.

At Kanyawara, P. F. recorded chimpanzee calls using a Sennheiser ME67 shotgun microphone and a Marantz Professional PMD661 solid‐state recorder. He recorded with a 44.1 kHz sampling frequency and a 16‐bit amplitude resolution. He obtained the recordings during continuous sampling of focal individuals (October 2010 to September 2011). In addition to recording all calls from the focal individual, he recorded any other vocal interactions between the focal and other individuals in the focal party. For each recording, he noted the identity of the caller who started a vocal bout, the identities of any other callers in a vocal bout, and the context of the vocalizations. He obtained calls in two contexts: in which the caller was either traveling or feeding (or arriving at a feeding site with visible food). Using the same selection criteria as Gombe, we obtained 111 calls from 7 Kanyawara males for acoustic analysis (Table 1).

### 2.3 Potential sampling biases

We evaluate the sources of bias using the STRANGE framework (Webster & Rutz, 2020). STRANGE stands for Social background; Trappability and self‐selection; Rearing history; Acclimation and habituation; Natural changes in responsiveness; Genetic make‐up; and Experience. In terms of social background and self‐selection, previous studies indicate that high‐ranking males call more frequently (Wilson et al., 2007), so they are more likely to be sampled (Table 1). We attempted to avoid overcontribution from any particular individual in our statistical analyses by performing multiple permutations on balanced and randomized subsets of the data (see Section 2.6), but some bias toward individuals that call more frequently might have been introduced due to needing a minimum number of recordings from each individual (see Section 2.2). Furthermore, the chimpanzee community sizes included in this study (Kasekela ~50 individuals, Mitumba ~30 individuals (Wilson et al., 2020), and Kanyawara ~54 individuals) are close to the median community size of 39.2 individuals observed in long‐term studies of wild chimpanzees (Wilson et al., 2014). In terms of rearing history, acclimation, and habituation, the chimpanzees at both Gombe and Kanyawara are wild, but were well habituated to observers at the time of recording. Additionally, since our studies were strictly observational, we did not subject chimpanzees to any invasive testing, thus mitigating any potential biases from acclimation, habituation, and experience. Natural changes in responsiveness due to seasons or timing could be sources of bias as chimpanzees produce pant‐hoots more frequently in the mornings (Wilson et al., 2007) and pant‐hoot production may vary with season depending on fruit availability (personal observation). While we followed the chimpanzees throughout the day and in all seasons, the sample is likely to contain more recordings from the mornings and from the wet season. Lastly, 3 out of 6 individuals at Kasekela were close kin (two brothers: F. U. and F. N. D. and their father: S. L.; Table 1) and none of the other individuals at any of the communities included were known to be close kin. If calls of genetically related chimpanzees are more similar, then calls of Kasekela individuals might appear different from other communities due to genetic similarity (Walker et al., in revision).

### 2.4 The pant‐hoot call

**Figure 1:**
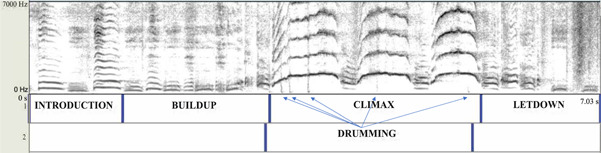
A spectrogram of a typical pant‐hoot call with the four phases and drumming labeled

The pant‐hoot is a complex call composed of multiple elements. Researchers typically divide pant‐hoots into four phases, each of which consists of one or more acoustically similar elements: (i) the introduction—inhaled and exhaled tonal elements (fundamental frequency F0: 300−600 Hz), (ii) the build‐up—shorter but more frequent exhaled tonal elements and noisy inhaled elements (F0: 200−500 Hz), (iii) the climax—loud tonal screams (F0: 800−2000 Hz) but often including other elements such as hoos and barks, (iv) the let‐down—short, build‐up‐like exhaled elements, decreasing in F0 (Figure 1, Sound S1) (Crockford et al., 2004; Mitani et al., 1992, 1999). Chimpanzees do not always produce all four of these phases when giving pant‐hoot calls. Sometimes during the call, chimpanzees hit tree buttresses with their feet (and rarely with their hands), producing drum‐like sounds (Arcadi & Wallauer, 2013).

Distinguishing these pant‐hoot phases can be difficult as the elements vary substantially in their acoustic structure within each phase. To address this ambiguity and to distinguish systematically among these phases, we proceeded as follows. We identified the exhaled elements in all phases as the elements that reached relatively higher maximum frequencies compared to elements preceding and succeeding them. To distinguish between the introduction and the build‐up phase, we defined the start of the build‐up as the first exhaled element of markedly shorter duration compared to the previous elements. The build‐up consisted of a series of elements with a similarly short duration. Next, to distinguish between the build‐up and the climax, we defined the start of the climax as the first exhaled element with a fundamental frequency greater than 500 Hz (see “500 Hz” rule Mitani et al., 1999). Next, to distinguish between the climax and the let‐down, we defined the end of the climax as the last tonal scream element. In cases where the climax phase did not include screams, we marked the end of the climax as the first element of a reduced fundamental frequency. The let‐down phase consisted of a series of these elements of a lower fundamental frequency. Some studies have identified several different kinds of elements within the climax phase (Crockford et al., 2004). Here we categorized climax elements as either scream or non‐scream elements that we could reliably differentiate.

### 2.5 Acoustic feature extraction

Given the structure of the pant‐hoots described above, acoustic analysis of pant‐hoots could be performed by measuring acoustic features in different ways. We extracted two main categories of acoustic features from the spectrogram representations of the calls: structural features and spectral features. Structural features describe the composition of the elements in different phases of the pant‐hoots and their temporal patterning. For these, we selected 25 acoustic features intended to be as similar as possible to those used in previous studies of chimpanzee dialects (Crockford et al., 2004; Mitani et al., 1992, 1999) (Table 2). Spectral features quantify the frequency and tonal structure of individual elements from the power spectrum. We measured these from selected specific elements: one build‐up element (24 features), and one climax element (25 features). We used semiautomatic measurements of acoustic features—a process involving manually chosen call elements used in automatic feature extraction—using Avisoft‐SASLab Pro v. 5.2 (Specht, 2004) and LMA (Fischer et al., 2013; Schrader & Hammerschmidt, 1997) (Table 3).

**Table 2:** Structural acoustic features manually measured using Praat v 6.1.15 that were used in this study

Structural acoustic features used in this study	Part of the pant‐hoot	Crockford et al. (2004)	Mitani et al. (1999)	Mitani et al. (1992)
Duration of the call (from build‐up to let‐down phases) (s)	Entire call	No	No	No
Presence of introduction phase*	Introduction	Yes	No	No
Presence of build‐up phase*	Build‐up	Yes	No	No
Number of build‐up exhalation elements	Build‐up	Yes	No	No
Number of build‐up elements in the first half of the build‐up	Build‐up	Yes	No	No
Number of build‐up elements in the second half of the build‐up	Build‐up	Yes	No	No
Duration of build‐up phase (s)	Build‐up	Yes	Yes	No
Rate of build‐up phase (elements/s)	Build‐up	Yes	Yes	Yes
Rate of first half of build‐up phase (elements/s)	Build‐up	Yes	No	No
Rate of second half of build‐up phase (elements/s)	Build‐up	Yes	No	No
Build‐up acceleration (rate of second half−rate of first half)	Build‐up	Yes	No	No
Presence of climax phase*	Climax	Yes	No	No
Total number of climax elements (including screams and non‐scream elements)	Climax	Yes	No	No
Number of screams in climax	Climax	Yes	No	No
Proportion of climax elements that are screams	Climax	Yes	No	No
Duration of climax phase (s)	Climax	No	No	No
Presence of let‐down phase*	Let‐down	Yes	No	No
Number of elements in let‐down phase	Let‐down	Yes	No	No
Structural acoustic feature(s) NOT used in this study				
Number of introduction elements	Introduction	Yes	No	No
Duration of introduction element	Introduction	No	Yes	No
Drumming related features**	Drumming	Yes	No	No

*Note*: We also indicate which features were used in other studies of chimpanzee dialects. Categorical variables are marked with *. Only numeric variables were used in the multivariate analyses including the PCAs and pDFAs since those techniques do not handle categorical variables.

Abbreviations: PCAs, principal component analysis; pDFAs, permuted discriminant function analysis.

**Drumming related variables were not included in the multivariate analysis due to small sample sizes. However, descriptive plots are included in the Supporting Information materials.

**Table 3:** Semiautomatically measured acoustic features using LMA from the selected build‐up and climax elements compared with other studies

Acoustic feature(s) used in this study	Crockford et al. (2004)	Mitani et al. (1999)	Mitani et al. (1992)
Duration of the element (ms)	No (for build‐up); yes (for climax)	Yes	Yes
Start, end, maximum, minimum, and mean fundamental frequency F0 (Hz)	No (for build‐up); yes (for climax)	No (start and end); yes (maximum, minimum, and mean)	No
Frequency range of F0 (Maximum F0−Minimum F0) (Hz)	No	Yes	Yes
Tonality measures: mean and maximum frequency difference between the original F0 curve and the floating average curve (Hz)	No (for build‐up); yes (for climax)	No	No
Location of maximum F0 relative to the duration ([1/duration] × location)	No (for build‐up); yes (for climax)	No	No
Factor of linear trend of F0 (measures if the F0 is rising, falling, or flat on average)	No (for build‐up); yes (for climax)	No	No
Mean and maximum deviations between F0 and linear trend line (Hz)	No (for build‐up); yes (for climax)	No	No
Start, end, maximum, minimum, and mean peak frequencies (Hz)	No (for build‐up); yes (for climax)	No	No
Peak frequencies with maximum and minimum amplitude (Hz)	No (for build‐up); yes (for climax)	No	No
Locations of maximum and minimum peak frequencies relative to the duration ([1/duration] × location)	No	No	No
Maximum difference between peak frequency values in successive time segments (Hz)	No (for build‐up); yes (for climax)	No	No
Mean and maximum wiener entropy coefficient (0−1; 1 = noise)	No	No	No
Acoustic feature(s) NOT used in this study			
Slope of F0 from start to maximum (Hz/ms)	Yes	No	No
Slope of peak frequency from start to maximum (Hz/ms)	Yes	No	No
Maximum F0 start F0 (Hz) and Maximum F0 minimum F0 (Hz)	Yes	No	No
F0 at midpoint of introduction element (Hz)	Yes	Yes	No
Peak frequency at midpoint of inhaled elements (Hz)	Yes	No	No
Peak frequency at midpoint of exhaled elements (Hz)	Yes	No	No
Peak frequency of inhaled−peak frequency of exhaled elements (Hz)	Yes	No	No
Ratio of F1/F2, the first and second formant frequencies	No	Yes	No
Bandwidth (Hz)	No	Yes	No

*Note*: Some acoustic features were not used in this study as they were not measured by the version of LMA available to us.

We extracted the acoustic features as follows. First, we measured structural features from the pant‐hoot phases by visually inspecting spectrograms of entire pant‐hoots using Praat version 6.1.15. We considered each phase separately and measured a set of acoustic features from each phase (Table 2). We present the visual summaries of these structural features of the pant‐hoots from the three communities in the Supporting Information: (Figures S3 [a–m]). Next, for the semiautomatic extraction of acoustic features, we chose one element from the build‐up phase and one element from the climax phase. From the build‐up phase, we chose the middle element in case of an odd number of build‐up elements, and the element immediately preceding the middle of the build‐up in case of an even number of elements (Mitani et al., 1999). From the climax phase, we chose the scream that reached the highest fundamental frequency in the spectrogram. To obtain appropriate frequency and time resolutions, we down‐sampled the sampling frequency to 24 kHz using Avisoft‐SASLab Pro, resulting in a frequency range of 12 kHz. Next, using Avisoft‐SASLab Pro, we created spectrograms with an FFT length of 1024 points, frame size of 100%, and Hamming window with an overlap of 93.75%. This resulted in a frequency resolution of 23 Hz, and a time resolution of 2.7 ms, which is sufficient to reveal tonal properties and extract acoustic features from build‐up and climax elements. We then imported the spectrograms in LMA and extracted acoustic features (listed in Table 3) using the harmonic cursor tool. We did not extract additional acoustic features from elements in the introduction and the let‐down phases as the introduction was not always recorded fully, and the let‐down exhibited high variability in the type of elements, making comparison among let‐downs difficult. We attempted to be consistent with previous studies by including as many acoustic features used in previous studies as possible (Tables 2 and 3). However, some acoustic features used in previous studies could not be measured using the software packages available to us. Nevertheless, we consider that the acoustic features we used should encompass the relevant range of variation in chimpanzee pant‐hoots, without loss of generality. To further facilitate comparisons with previous studies, we report means and standard deviations of acoustic features found to have community specific differences in previous studies in the Supporting Information: Table S1.

### 2.6 Statistical analysis

To confidently make conclusions about whether chimpanzees have community‐specific dialects that are an outcome of vocal learning, we need to control for confounding factors, which we examine using directed acyclic graphs (DAGs) (McElreath, 2020). DAGs portray our assumptions of causal relationships among variables. Based on these biologically informed assumptions, DAGs allow us to (i) identify confounding causal paths that may cause spurious statistical associations between variables, (ii) identify causal paths that may mask real causal relationships. Thus, DAGs allow us to make biologically informed decisions about which confounding variables to control in our statistical analyses.

**Figure 2:**
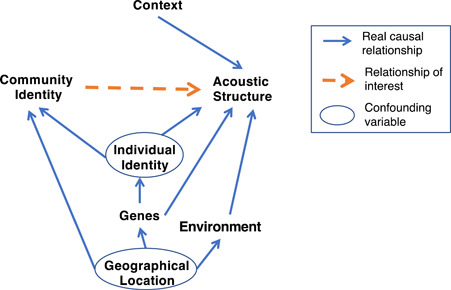
Directed acyclic graph of the assumed causal relationships among relevant variables

Figure 2 portrays our assumed causal relationships as follows: the geographical location of a community can affect the environmental conditions (because environmental features such as forest structure may cause locations to vary in habitat acoustics), community identity (because communities are in part defined based on geographical proximity), and genetics of the chimpanzees (because geographically closer chimpanzees are more likely to be genetically related). Genetic similarity and environmental conditions such as habitat acoustics and sound environment can in turn affect the acoustic structure of vocalizations (Mitani et al., 1999). Furthermore, genetics affects individual identity, and individual identity may affect both acoustic structure and community identity, because communities are defined as a group of individuals that live within the same territory. Lastly, context may affect acoustic structure (Fedurek et al., 2016; Notman & Rendall, 2005; Uhlenbroek, 1996). We used this DAG, to identify minimally sufficient adjustment sets for assessing the relationship of interest, that is, community identity and acoustic structure. A minimally sufficient adjustment set of variables is a list of variables that are sufficient to control for estimating, in an unbiased way, the statistical association of two variables in a DAG. The *adjustmentSets* function in the R‐package dagitty prints a list of all minimally sufficient adjustment sets (Textor et al., 2016). This package identified the sets (individual identity, geographical location) and (environment, genetics, individual identity) as the minimally sufficient adjustment sets to assess the relationship between community identity and acoustic structure. Hence, we need to either control for individual identity and geographical location, or environment, genetics, and individual identity. Since we did not measure environmental variables or genetics, we could not control for those. However, we could obtain an unbiased association between community identity and acoustic structure by controlling for individual identity and geographical location. We therefore controlled for geographical location by testing for differences between calls from neighboring communities and compared them with the geographically distant Kanyawara community. Next, we controlled for individual identity using the permuted discriminant functions analysis (pDFA) procedure. The pDFA procedure is used to test for differences in a factor of interest (a.k.a. test factor) while controlling for a confounding factor (a.k.a. control factor) (Mundry & Sommer, 2007). We needed to control for individual identity not only to close confounding “backdoor” pathways, but also to account for the nonindependence of data points due to there being multiple recordings from the same individual. We describe the pDFA procedure in more detail in the next section. Lastly, while context is not a confound opening any “backdoor” paths based on our DAG, context is a precision covariate that may affect the relationship of interest (community ID to acoustic structure) (Laubach et al., 2021). Controlling for a precision covariate could improve the precision of our model estimates and prevent any masking of the relationship of interest (*ibid.*). Hence, we control for context for each set of acoustic features for which there exist differences between contexts.

### 2.7 Description of the pDFA procedure

The pDFA procedure improves upon traditional discriminant functions analysis (DFA) technique by allowing two‐factor designs. The traditional DFA technique handles only one factor at a time and is known to inflate group differences in two‐factorial designs with a confounding factor (individual identity is among the most common confounding factors in similar studies [Mundry & Sommer, 2007]). The pDFA procedure allows two‐factor designs by performing a permutation test on the classification accuracy of DFA. The permutation test tests if the observed classification accuracy of DFA is significantly higher than expected by chance while accounting for the accuracy inflating effect of a confounding factor. The procedure works as follows. First, the procedure samples a specified number of balanced and randomized data sets from the original data set. It randomizes the labels of the test factor based on the combinations of different categories of test and control factors. It performs the balancing such that there is the same number of observations from each category of the test factor. Next, it performs a traditional DFA on each of these randomized data sets and obtains a distribution of classification accuracies of these DFAs. The observations left out due to balancing are used for cross‐validation to obtain the out‐of‐sample, cross‐validated classification accuracies. The distribution of classification accuracies of randomized data sets describes the probabilities of obtaining particular classification accuracies using a traditional DFA just based on chance. The expected value from this distribution is compared with the classification accuracy obtained on the original data set (observed classification accuracy) to obtain a *p* value for the permutation test. In other words, this distribution provides an estimate of the inflation in classification accuracy of the test factor caused by the confounding effect of the control factor.

### 2.8 Analysis steps

We performed the pDFAs with 1000 permutations (i.e., 1000 randomized data sets including the original data set) in each of our analyses and used an alpha level of *α*= 0.05 on the cross‐validated classification accuracy to infer a significant difference. The test factors of interest in our study were context, community identity, and individual identity. The control factors were context and individual identity, depending on the analysis. For different test factors in the pDFA, we had to consider the data designs to ensure proper randomization and balancing of the permuted data sets. In our study, two design situations occurred: crossed and nested. A crossed design occurs when all the categories of the test factor are recorded in all categories of the control factor. So, a pDFA with crossed design could only be used in testing for differences in context by only including individuals recorded in both contexts. While testing for differences in community identity and individual identity, a nested design occurs. In a nested design, the categories of the control factor are nested within categories of the test factor, or the categories of the test factor are nested within some other factor known as the restriction factor. Since individual identity is nested within community identity, a nested design occurs when testing for differences in the community or individual identities.

We performed all statistical analyses in R version 4.0.2 using RStudio version 1.3.1093. The pDFAs were carried out using a set to R functions provided by R. Mundry. These functions implement the pDFA procedure and are built on top of the *lda* function in the R package MASS (Ripley et al., 2020) that is used to perform traditional DFAs. We performed four different analyses for different kinds of acoustic features: (i) structural features (Table 2); (ii) build‐up element features (Table 3); (iii) climax scream features (Table 3); and (iv) all features combined (Tables 2 and 3). We tested for differences in context, community identity, and individual identity in each of these four types of acoustic features. For each kind of acoustic feature set, we tested for context before performing other analyses to determine whether context was a precision covariate (Laubach et al., 2021) that needed to be statistically controlled for in the subsequent analyses (refer to the DAG logic in Section 2). If we found statistically significant differences in the context in any acoustic feature set, we controlled for the context by stratifying the data and considering calls from only one context at a time in separate analyses (Crockford et al., 2004). If we did not find any significant effect of context on an acoustic feature set, we did not need to control for context. To control for geographical differences, we performed two separate analyses for each kind of acoustic feature set. Following Crockford et al. (2004), we first investigated the acoustic structure of pant hoots from the two neighboring communities of Gombe, where maximal differences were expected. To then compare the two Gombe communities to a geographically distant community, we ran pDFAs including all three communities. To control for individual differences, we used individual identity as the control factor in each of the pDFAs when testing for context and community identity. When testing for individual differences using individual identity as the test factor, we used community identity as the restriction factor. When a restriction factor is added in the pDFA, the randomization process is done while accounting for the fact that the test factor is nested within the restriction factor. To avoid overfitting, we ensured that only as many, or fewer acoustic features are used to perform the DFAs as there are observations in the category of the test factor with the fewest observations. To avoid multicollinearity and to reduce the number of acoustic features while accounting for most variation contained in different acoustic features, we used PCA on the acoustic features. We used the scores of each observation on the principal components as the features to be used in the DFAs. To choose the number of principal components to include, we used two heuristics. First, we used as many principal components as there were number of observations in that category of the test factor with the fewest observations or as many principal components that explained 90% of the variation, whichever was smaller. Limiting the number of principal components to those that explained 90% of the variation allowed us to avoid including too many components of little explanatory power when including many more components was possible in the pDFA design. Second, since no heuristic is perfect in all circumstances (Jolliffe, 2002), we used an additional heuristic to ensure the stability of results. We verified the consistency of the results of the pDFAs over different numbers of principal components selected using Cattell's scree test (Cattell, 1966). Using this heuristic, we chose the number of principal components by identifying the “elbows” in the scree plot of variances explained against the number of the principal component.

For each acoustic feature set used in the analyses, we chose a subset of recordings based on the following criteria. For structural features, we first removed acoustic features that had too many missing values for sufficient statistical power. These mainly included acoustic features related to drumming, as only 18% of the recorded pant‐hoots had drumming (Table 2, Supporting Information: Figures S3 [l–m]). After that, we removed categorical features that indicated the presence or absence of the four phases as categorical features are not handled by DFAs. Next, we removed the cases that had missing values in any of the remaining 14 acoustic features. While the categorical features were eliminated, the information contained in them was included in other features that indicated the number of elements in each phase. A value of 0 in those features would indicate absence of a phase, whereas a non‐zero value would indicate the presence. For the build‐up feature set, we only included pant‐hoots that included the build‐up phase. Similarly, for the climax feature set, we only included pant‐hoots that included the climax phase. And lastly, while including all the acoustic features simultaneously (structural, build‐up, and climax features), we only included pant‐hoots that had both build‐up and climax phases.

The pDFA is our omnibus test that warrants further post hoc tests in case it revealed significant differences. To perform post hoc tests, we used the *repDFA* function written by C. Neumann (Berthet et al., 2017; Neumann, 2020) followed by generalized linear mixed models (GLMMs). *repDFA* function allows us to identify the key variables that discriminate the test factor. This function creates 1000 balanced data sets in crossed designs, reruns 1000 DFAs and records the variable that had the highest coefficient on the first and second linear discriminant functions in each of those DFAs. Variables that have the highest coefficient in many of those permutations are arguably the most important in discriminating the test factor. For nested designs, we modified Neumann's function and wrote a new function called *repDFA_nested* (available on GitHub). This function modified *repDFA* such that it created balanced data sets by randomly sampling with replacement the same number of recordings for each individual in the analysis. Next, we tested the significance of the individual variables identified with *repDFA* and *repDFA_nested* using GLMMs. We controlled for individual identity in the GLMMs by including individual IDs as random intercepts and adjusted the *p* values for multiple comparisons using Benjamini−Hochberg adjustment method.

### 2.9 Ethical note

The research reported in this paper is based on data collected noninvasively from free‐ranging chimpanzees. The Institutional Animal Care and Use Committee at the University of Minnesota did not require a review due to the purely observational nature of the research. Research at Gombe National Park was performed with approval from the Tanzania Wildlife Research Institute and the Tanzania Commission for Science and Technology and adhered to additional ethical guidelines set by the Jane Goodall Institute.

Research at Kibale National Park was approved by the Department of Psychology Ethics panel at the University of York and permission to conduct the study was granted by the Ugandan Wildlife Authority and the Ugandan National Council for Science and Technology. The study complied with the laws of Uganda.

## 3 RESULTS

### 3.1 Differences in pant‐hoots between contexts

**Figure 3:**
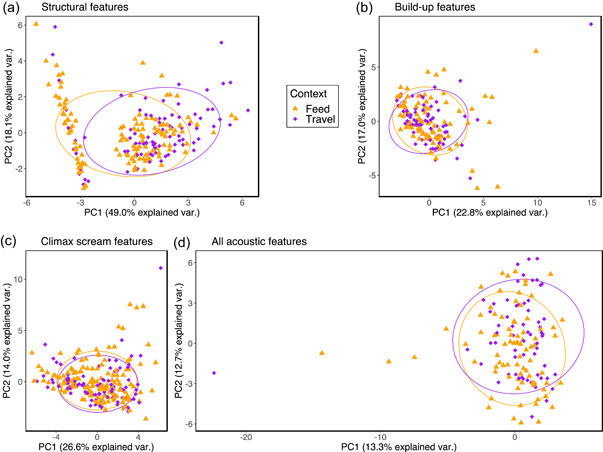
Principal components plots with the 68% normal data ellipses containing 68% of the data points included for each context. (a) Principal components analysis performed on structural features. Pant‐hoots given in different context separate over PC1. (b) Principal components analysis performed on acoustic features of the selected build‐up element. (c) Principal components analysis performed on acoustic features of the selected climax element. (d) Principal components analysis performed on all acoustic features simultaneously from all three communities. (b), (c), and (d) reveal strong overlap between contexts. PC1, principal component 1; PC2, principal component 2.

To ascertain if we needed to control for context in our main analyses regarding acoustic differences in pant hoot structure as a function of community, we started by examining if context affected any of our acoustic feature sets. We found a statistically significant difference in the structural acoustic features (Table 2) between feeding and traveling contexts after controlling for individual identity (pDFA with structural features, observed classification accuracy: 63.9% vs. expected by chance: 51.7%; *p* = 0.044, Table 4). Using the *repDFA* function, we identified principal component 6 (PC6) to be the best discriminator of the contexts in 716 out of the 1000 DFAs followed by principal component 1 (PC1) in 191 out of the 1000 DFAs. PC6 loaded most heavily on the number of let‐down elements and build‐up acceleration had the second highest loading. PC1 loaded most heavily on the number of build‐up elements but other features of the build‐up had comparable loadings. These features were: duration of the build‐up phase, rate of the build‐up phase, number of elements in the first and second half of the build‐up, and rate of the first and the second half of the build‐up. Further, the principal components plot made by performing PCA on the structural features shows a distinct band of calls given mostly in feeding contexts (Figure 3a). Principal component 2 (PC2) explained the most variance in this band and it loaded most heavily on the number of climax elements. These were pant‐hoots with a greater than average number of climax elements, and which did not have a build‐up phase. We performed significance tests for the highest loading features of these three important components using Poisson GLMMs and controlled for individual identity by including it as a random effect in the models. All three of these acoustic features were statistically significantly different between contexts. Travel pant‐hoots had a (i) greater number of let‐down elements, *β* (Travel) = 0.57, Benjamini−Hochberg adjusted *p* value = 3.8e−07; (ii) greater number of build‐up exhalation elements, *β* (Travel) = 0.34, Benjamini−Hochberg adjusted *p* value = 4.8e−06; and (iii) lower number of climax elements, *β* (Travel) = −0.27, Benjamini−Hochberg adjusted *p* value = 1.8e−03, compared to feeding pant‐hoots (Supporting Information: Figures S2 [a–c], Table 5). Hence, we conclude that the number of elements in the build‐up, the climax, and the let‐down phases potentially encoded contextual information.

**Table 4:** Summary of the results from the pDFAs with context as the test factor and individual identity as the control factor for different types of acoustic features

Acoustic features used	Control factor	Number of individuals included in both contexts	Median number of calls per individual in each context (range)	Total number of calls used	Observed cross‐validated classification accuracy (expected value)	*p* Value for cross‐validated classification accuracy
Communities from Gombe						
Structural (Table 2)	Individual	5	Feed: 7 (3−25)	82	63 (55.9)	0.291
			Travel: 4 (3−9)			
Build‐up (Table 3)	Individual	5	Feed: 6 (3−20)	66	52.8 (50.9)	0.382
			Travel: 4 (3−7)			
Climax (Table 3)	Individual	6	Feed: 7.5 (3−26)	100	50.1 (49.4)	0.394
			Travel: 5.5 (3−7)			
Entire call (Tables 2 and 3)	Not performed due to low sample sizes of individuals recorded in both contexts					
All communities						
Structural (Table 2)	Individual	11	Feed: 7 (3−25)	183	63.9 (51.7)	0.044*
			Travel: 6 (3−12)			
Build‐up (Table 3)	Individual	9	Feed: 6 (3−20)	121	51.9 (49.6)	0.376
			Travel: 6 (3−12)			
Climax (Table 3)	Individual	12	Feed: 7.5 (3−26)	203	52.2 (50.2)	0.34
			Travel: 6.5 (3−12)			
Entire call (Tables 2 and 3)	Individual	6	Feed: 6 (3–9)	77	50.8 (47.7)	0.37
			Travel: 6.5 (3−12)			

*Note*: We indicate the number of individuals included in the test factor, that is, in both feeding and traveling contexts, the range of number of calls per individual and the total number of calls considered for each of the analyses.

Abbreviation: pDFAs, permuted discriminant function analysis.

*statistically significant at p < 0.05.

**Table 5:** Structural acoustic features showing differences between contexts

Acoustic variable	Context	$\bar{x}\pm \mathrm{SD}$	*β* (travel)	*p* Value
Number of let‐down elements	Feed	1.26 ± 1.36	0.57	3.8e−07
	Travel	2.34 ± 2.59		
Number of build‐up exhalation elements	Feed	3.21 ± 3.2	0.34	4.8e−06
	Travel	5.31 ± 4.13		
Number of climax elements	Feed	3.18 ± 2.34	−0.27	1.8e−03
	Travel	2.63 ± 2.34		

Further, we found no differences in the contexts in other types of acoustic features after controlling for individual identity among the contiguous communities or all communities taken together. Cross‐validated *p* values for pDFA performed on all communities with (i) build‐ups: *p* = 0.38, (ii) climax screams: *p* = 0.34, and (iii) all acoustic features simultaneously: *p* = 0.37. Cross‐validated *p* values for pDFA performed on communities within Gombe with (i) structural features: *p* = 0.29, (ii) build‐ups: *p* = 0.38, and (iii) climax screams: *p* = 0.39 (Table 4). Figure 3b−d show the overlap between contexts in these acoustic features in a multidimensional space. Given that context is confounding only when it has a significant effect (refer to DAG logic in Section 2), we controlled for context when testing for differences in structural features alone and not when testing for differences in other types of acoustic features in the subsequent analyses.

### 3.2 Differences in pant‐hoots among communities of chimpanzees

**Figure 4:**
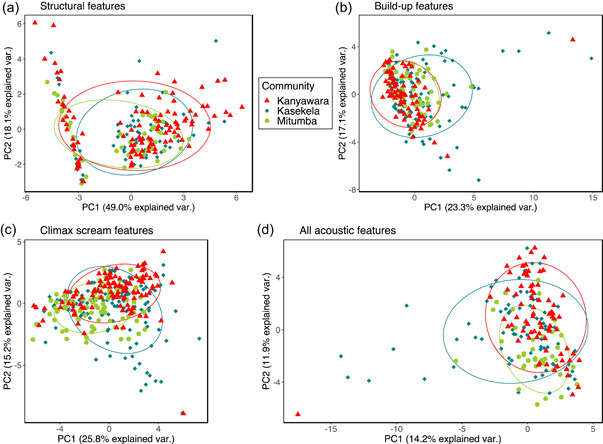
Principal components plots with the 68% normal data ellipses containing 68% of the data points included for each community. (a) Principal components analysis performed on structural features. (b) Principal components analysis performed on acoustic features of the selected build‐up element. (c) Principal components analysis performed on acoustic features of the selected climax element. Kasekela and geographically distant Kanyawara communities separate to some extent over PC2. (d) Principal components analysis on all acoustic features simultaneously from all three communities. (a), (b), and (d) reveal strong overlap among communities. PC1, principal component 1; PC2, principal component 2.

Figure 4a–d show the clusters of the three communities in multidimensional spaces of the structural features, build‐ups, climaxes, and all features taken simultaneously. These show strong overlap among communities, suggesting a lack of community‐level differences that is confirmed by the pDFAs, with one exception: a statistically significant difference in acoustic features of the climax scream among the communities when we included the geographically distant Kanyawara community in the analysis (pDFA on climaxes of all communities, observed classification accuracy: 54% vs. expected: 40.8%; *p* = 0.016, Table 6). These features of the climax screams did not differ significantly between the two neighboring communities at Gombe, but the relatively low *p* value indicates these features may warrant further investigation (observed classification accuracy: 70.7% vs. expected: 58%; *p* = 0.089, Table 6). Furthermore, these features did not differ between the pair Kasekela‐Kanyawara (observed classification accuracy: 88% vs. expected: 77.1%; *p* = 0.18) or between the pair Mitumba‐Kanyawara (observed classification accuracy: 68.1% vs. expected: 57.2%; *p* = 0.13). Hence, we did not use the *repDFA_nested* function to test which acoustic features were important in the 3‐community analysis. Considering that the DFA could be sensitive to outliers (Mundry & Sommer, 2007), we checked for the consistency of the results after removing outliers. The patterns remain similar after the removal of outliers (See Supporting Information: Figures S2 [a–b]).

**Table 6:** Summary of the results from the pDFAs with community identity as the test factor for different types of acoustic features

Acoustic features used	Control factor	Number of individuals included per community	Median number of calls per individual in each community (range)	Total number of calls used	Observed cross‐validated classification accuracy (expected value)	*p* Value for cross‐validated classification accuracy
Communities from Gombe						
Structural (Table 2)	Individual (calls included from both contexts)	Kasekela: 6	Kasekela: 8 (4−21)	103	53 (56)	0.639
		Mitumba: 3	Mitumba: 7 (4−34)			
Structural (Table 2)	Individual (calls only from feed context)	Kasekela: 3	Kasekela: 7 (5−18)	61	63.84 (58.77)	0.368
		Mitumba: 3	Mitumba: 3 (3−25)			
Structural (Table 2)	Individual (calls only from travel context)	Kasekela: 6	Kasekela: 4 (3−6)	39	44.41 (51.34)	0.725
		Mitumba: 2	Mitumba: 6.5 (4−9)			
Build‐up (Table 3)	Individual	Kasekela: 6	Kasekela: 13 (6−33)	146	58.5 (53.5)	0.255
		Mitumba: 4	Mitumba: 9 (5−24)			
Climax (Table 3)	Individual	Kasekela: 6	Kasekela: 15.5 (8−34)	199	70.7 (58)	0.089
		Mitumba: 5	Mitumba: 9 (8−41)			
Entire call (Tables 2 and 3)	Individual	Kasekela: 5	Kasekela: 11 (6−28)	115	59.6 (54.6)	0.272
		Mitumba: 5	Mitumba: 5 (3−19)			
All communities						
Structural (Table 2)	Individual (calls included from both contexts)	Kasekela: 6	Kasekela: 8 (4−21)	212	35.4 (39.7)	0.729
		Mitumba: 3	Mitumba: 7 (4−34)			
		Kanyawara: 7	Kanyawara: 14 (8−26)			
Structural (Table 2)	Individual (calls only from feed context)	Kasekela: 3	Kasekela: 7 (5−18)	126	37.54 (41.37)	0.655
		Mitumba: 3	Mitumba: 3 (3−25)			
		Kanyawara: 7	Kanyawara: 7 (3–23)			
Structural (Table 2)	Individual (calls only from travel context)	Kasekela: 6	Kasekela: 4 (3−6)	82	32.5 (36.28)	0.676
		Mitumba: 2	Mitumba: 6.5 (4−9)			
		Kanyawara: 6	Kanyawara: 7 (3−12)			
Build‐up (Table 3)	Individual	Kasekela: 6	Kasekela: 13 (6−33)	222	45 (37.6)	0.08
		Mitumba: 4	Mitumba: 9 (5−24)			
		Kanyawara: 7	Kanyawara: 10 (6−20)			
Climax (Table 3)	Individual	Kasekela: 6	Kasekela: 15.5 (8–34)	310	54.0 (40.8)	0.016*
		Mitumba: 5	Mitumba: 9 (8−41)			
		Kanyawara: 7	Kanyawara: 14 (8−26)			
Entire call (Tables 2 and 3)	Individual	Kasekela: 5	Kasekela: 11 (6−28)	191	51.3 (42.2)	0.079
		Mitumba: 5	Mitumba: 5 (3−19)			
		Kanyawara: 7	Kanyawara: 10 (6−20)			

*Note*: We indicate the control factor, the number of individuals included in the test factor, that is, from each community, the range of number of calls per individual and the total number of calls considered for each of the analyses. We used context as a control factor only in case of structural features since there was a difference between contexts only in structural features.

Abbreviation: pDFAs, permuted discriminant function analysis.

*statistically significant at p < 0.05.

Additionally, we observed no differences between the contiguous communities, or among all three communities, in the structural features (controlled for individual identity and context), acoustic features of the build‐ups, or all acoustic features considered together (Table 6).

### 3.3 Differences in pant‐hoots among individuals

We observed statistically significant differences among the individuals in the structural features, acoustic features of the climax screams, and all acoustic features taken simultaneously. This was true when all communities were taken together as well as when the geographically adjacent communities of Gombe were assessed separately (Table 7). However, the individuals could not be separated based on acoustic features of the selected build‐up elements in any setting (pDFA on build‐up features of all communities: *p* = 0.18, and Gombe: *p* = 0.15; Table 7).

**Table 7:** Summary of the results from the pDFAs with individual identity as the test factor for different types of acoustic features

Acoustic features used	Control or restriction factor	Number of individuals included	Median number of calls per individual (range)	Total number of calls used	Observed cross‐validated classification accuracy (expected value)	*p* Value for cross‐validated classification accuracy
Communities from Gombe						
Structural (Table 2)	Community (restriction factor)	11	13 (4−41)	171	24 (10.3)	0.001*
Structural (Table 2)	Context (control factor)	Not performed due to low sample sizes of individuals recorded in both contexts				
Build‐up (Table 3)	Community (restriction factor)	10	12 (5−33)	146	16.1 (12.1)	0.15
Climax (Table 3)	Community (restriction factor)	11	12 (8−41)	199	24.2 (13.2)	0.006*
Entire call (Tables 2 and 3)	Community (restriction factor)	10	10 (3−28)	115	23.5 (13.2)	0.024*
All communities						
Structural (Table 2)	Community (restriction factor)	18	13.5 (4−41)	280	19.5 (6.9)	0.001*
Structural (Table 2)	Context (control factor)	7	14 (9−34)	119	35.8 (24.7)	0.043*
Build‐up (Table 3)	Community (restriction factor)	17	11 (5−33)	222	10.6 (8.2)	0.18
Climax (Table 3)	Community (restriction factor)	18	14 (8−41)	310	20.1 (9.4)	0.001*
Entire call (Tables 2 and 3)	Community (restriction factor)	17	10 (3−28)	191	14.4 (7.6)	0.007*

*Note*: We used community ID as the restriction factor except when using context as a control factor. We indicate the number of individuals included, the range of number of calls per individual and the total number of calls considered for each of the analyses.

Abbreviation: pDFAs, permuted discriminant function analysis.

*statistically significant at p < 0.05.

**Figure 5:**
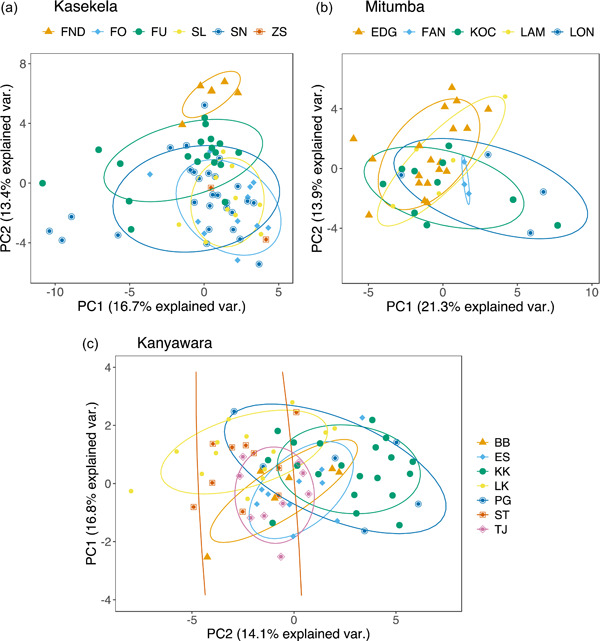
Principal components plots with the 68% normal data ellipses containing 68% of the data points included for each individual. Principal components analysis was performed on the structural features as well as features of the selected build‐up and climax elements simultaneously from the three communities. The 68% normal data ellipses revealed a lower overlap compared to community identity and context. Plot for (a) Kasekala. Some individuals formed distinct clusters over PC2. (b) Mitumba. Some individuals formed distinct clusters over a combination of PC1 and PC2. (c) Kanyawara. Some individuals formed distinct clusters over PC2 and others over PC1. PC1, principal component 1; PC2, principal component 2.

Figure 5a–c show the differences among individuals of the three communities in the multidimensional space all acoustic features taken simultaneously. In Kasekela, calls from the individuals FND, FU, and the pair FO and SL separate over PC2. While calls from FO, SL, and SN overlap, SN could be differentiated to some extent on PC1 (Figure 5a). In Mitumba, while calls from the individuals EDG and LAM overlap, they could be differentiated from KOC, LON, and FAN from a combination of PC1 and PC2 values (Figure 5b). In Kanyawara, calls from the individuals BB, ES, and TJ separate from LK on PC1 and from PG and KK on PC2 (Figure 5c).

For the structural features, the *repDFA_nested* function identified PC2 and principal component 7 (PC7) to have the highest loadings on both discriminant function 1 (PC2 higher than PC7) and discriminant function 2 (PC7 higher than PC2). Combined they had the highest loadings on discriminant function 1 in 619 out of 1000 DFAs and on discriminant function 2 in 423 out of 1000 DFAs. Top three acoustic features with the highest loadings on PC2 were the number of climax elements, build‐up to let‐down duration, and the duration of climax. And on PC7, the number of climax screams, build‐up to let‐down duration, and the duration of climax loaded the highest. For the climax features, the *repDFA_nested* function identified PC1 to have the highest loading on discriminant function 1 in 860 out of 1000 DFAs and principal component 3 (PC3) to have the highest loading on discriminant function 2 in 842 out of 1000 DFAs. The top three acoustic features with the highest loadings on PC1 were mean F0, maximum F0, and frequency range of F0. On PC3, the top three acoustic features were minimum F0, minimum peak frequency, and start frequency of F0. Lastly, when all features were taken together, PC2 loaded the highest on 945 out of 1000 DFAs. The top three acoustic features that loaded the highest on PC2 were maximum F0, mean F0, and frequency range of F0. This confirmed the findings above and also suggested that the strongest individual signal was in the acoustic features of the fundamental frequency in the climax scream. We do not report the results from the GLMMs for these acoustic features as differences among specific individuals are not the focus of this study. However, we observed statistically significant differences in some (but not all) pairs of individuals in each of these acoustic features suggesting that some individuals could be identified with more certainty than others. We can see this reflected in the low classification accuracies in the pDFAs (Table 7).

## 4 DISCUSSION

Our analysis of multiple acoustic features of chimpanzee pant‐hoots found that pant‐hoots could not be distinguished reliably based on the community identity, but instead reflected individual identity and potentially encoded some contextual information. The pant‐hoots differed among the communities in only one type of acoustic features (the acoustic features of the climax scream) in the omnibus pDFA, and only when we included the geographically distant Kanyawara community in the analysis. However, we did not find statistically significant pairwise differences among the communities in post hoc comparisons. Most importantly, we did not observe a statistically significant difference in the climax screams of the geographically adjacent communities of Gombe. We also did not observe any differences among the communities in either the structural features, the build‐up features, or when taking all the acoustic features simultaneously. The pant‐hoots differed most substantially among individuals, irrespective of the inclusion of the geographically distant community in our analyses. The acoustic features of the climax scream element and the structural acoustic features distinguished the individuals, whereas the acoustic features from the build‐up element alone did not. Collectively, our findings indicate that individual differences are more prominent than group differences in the acoustic structure of chimpanzee pant‐hoots.

We found that the context of the vocalization could be identified from some structural acoustic features but not from any other kind of acoustic features. Within the structural features, the number of climax elements was higher in feeding contexts and the number of let‐down elements as well as build‐up elements was higher in traveling contexts. Our results support the findings of Clark and Wrangham (1993), Fedurek et al. (2016), and Notman and Rendall (2005) in finding an association of the let‐down phase with the context of the pant‐hoot. All of them observed a greater number of pant‐hoots with let‐down components in traveling contexts, which is a finding consistent with our findings of observing a greater number of let‐down elements in traveling contexts. However, we did not have sufficiently detailed behavioral data to distinguish food arrival pant‐hoots separately, and hence, we could not confirm the finding of Clark and Wrangham (1993), that a higher proportion of pant‐hoots with let‐downs occurred in the context of arrival at a food source. We further observed two more differences that have not been reported previously. First, we found that pant‐hoots given in feeding contexts had more climax elements. Second, we observed a higher number of build‐up elements in travel context. Furthermore, we found no differences between the contexts in other acoustic features that describe the tonal properties of the build‐up and climax elements. Uhlenbroek (1996) described different types of pant‐hoots based on their tonal and spectral properties: a “wail‐like” pant‐hoot is a pant‐hoot with clear harmonic structure and a power spectrum with clear peaks; a “roar‐like” pant‐hoot is a noisy pant‐hoot lacking a clear harmonic structure and a more evenly distributed power spectrum (Uhlenbroek, 1996). Notman and Rendall (2005) found that pant‐hoots given in traveling contexts were more “roar‐like” and those given in feeding contexts were more “wail‐like.” Since we found no context differences in the acoustic features related to the tonal properties, fundamental frequency, noise, or peak frequency, we could not confirm these findings from either Uhlenbroek (1996) or Notman and Rendall (2005). Our results indicate that a more fine‐grained differentiation of contexts while recording pant‐hoots may be needed to distinguish arrival pant‐hoots as well as pant‐hoots from other contexts such as resting, grooming, displaying. Additionally, our findings suggest that future studies should pay special attention to the structural features whenever the context of pant‐hoot production is relevant to the analysis.

In contrast to communities and contexts, we found substantial differences among individuals. Individuals differed in structural features and in climax scream features, but not in build‐up element features. When all features were taken together, we observed the strongest differences in the climax scream features. The temporal properties that revealed greatest individual distinctiveness were duration of the climax phase, duration from build‐up to let‐down, and number of climax elements and screams. The spectral acoustic features showing the greatest individual differences were acoustic features related to the fundamental frequency F0. Specifically, the start, minimum, maximum, and mean F0, frequency range of F0, and minimum peak frequency were the features with the strongest individual level signal. Some of these acoustic features that correlated with individual differences were consistent with those identified by Crockford et al. (2004): maximum F0 and minimum peak frequency. Additionally, our results are consistent with results from previous studies by Mitani and colleagues (Mitani & Brandt, 1994; Mitani et al., 1999). Mitani and Brandt (1994) found that the principal component that explained the most variance among individuals loaded most highly in acoustic features of the fundamental frequency F0 including, start, minimum, maximum, and mean F0. Similarly, Mitani et al. (1999) found significant individual differences in the minimum, maximum, and mean F0, and the frequency range of F0. Lastly, we observed no differences among individuals in the spectral features of the build‐up elements. This is contrary to findings from previous studies (Fedurek et al., 2016; Mitani et al., 1999) that found associations of build‐up features with individual identity. However, Fedurek et al. (2016) found only modest associations of build‐up features with identity and Mitani et al. (1999) mainly considered temporal properties (structural features) of the build‐ups and did not consider spectral features other than the frequency. Hence, we may have failed to detect an association due to sampling variation or choice of acoustic features.

Our findings contrast with those of previous studies looking at community‐specific acoustic differences in pant hooting (Crockford et al., 2004; Marshall et al., 1999; Mitani et al., 1992). In the first study reporting vocal dialects in chimpanzees, Mitani et al. (1992) found differences in geographically distant communities of Gombe and Mahale National Parks. Mitani et al. (1999) subsequently reassessed these findings but still found differences between geographically distant and Mahale and Kibale National Parks. In contrast to Crockford et al. (2004), we did not observe any significant differences among neighboring communities. The consistencies and inconsistencies of our results with previous studies reveal several insights and raise new questions. Consistent with previous studies, our study confirms the individual distinctiveness of chimpanzee pant‐hoots in both spectral and temporal properties. Our study also found some differences in the temporal properties of pant‐hoots given in feeding and traveling contexts, confirming the possibility of some contextual encoding. In terms of community‐specific differences, we could not confirm previous studies of geographic variation in chimpanzees. However, vocalizing vertebrates often lack geographic variation in some vocalizations. For example, even in a species that exhibits extensive variation in vocalizations and vocal learning such as humpback whales, allopatric populations lack geographic variation in some call types (Fournet et al., 2018). Non‐song‐learning species of birds such as Barred Owls (*Strix varia*) (Odom & Mennill, 2012), Thick‐billed parrots (*Rhynchopsitta pachyrhyncha*) (Guerra et al., 2008), and doves (*Streptopelia* sp.) (De Kort et al., 2002) also lack geographic variation in many calls. Such instances of a lack of learned signals could be explained by genetic similarities and hybridization. For instance, loud calls of gibbon (*Hylobates* sp.) hybrids are not learned from parents and instead exhibit strong genetic inheritance (Brockelman & Schilling, 1984). Our failure to find evidence for community‐specific signatures in chimpanzees could reflect features peculiar to Gombe chimpanzees. Alternatively, it may be the case that previous findings of differences among chimpanzee communities resulted from statistical artifacts.

In chimpanzees, several community‐specific peculiarities can lead to differential selection pressures for community‐specific vocalizations. For example, (i) a recent history of intergroup violence could lead to a greater selection pressure for community‐specific vocalizations to facilitate identifying own community versus neighbors. There is a history of lethal intergroup violence in Gombe (Wilson et al., 2004), Kibale (Watts et al., 2006), as well as in Taï chimpanzees studied by Crockford et al. (2004) and (Boesch et al., 2008). However, Gombe chimpanzees have experienced a higher rate of intercommunity killings (Boesch et al., 2008; Wilson et al., 2004), suggesting that the selection for community‐specific vocalizations should be at least as strong as that for Taï chimpanzees, if not higher. (ii) Stability of hierarchy and strength of affiliative bonds in the community promote vocal convergence (Fedurek, Machanda, et al., 2013; Mitani & Brandt, 1994; Mitani & Gros‐Louis, 1998) and thus could create positive selection pressure for community‐specific vocalizations. In Gombe, within‐community bonds are likely stronger in the Kasekela community, which has more maternal brothers (Bray & Gilby, 2020) and closer overall genetic relatedness among males (Walker et al., in revision) compared to Mitumba, which has fewer brothers and higher within‐community violence (Massaro et al., 2021). More data are needed to accurately test if social bonds affect vocal convergence across field sites. (iii) A larger community size may lead to a greater selection pressure for community‐specific signatures as it becomes more difficult to keep track of individuals. All communities in this study and in Crockford et al. (2004) were moderate in size, median community size ±1SD: 39.2 ± 29.9 (Wilson et al., 2014), so the difference between our results and those of Crockford et al. (2004) are unlikely to result from differences in community size.

Another possibility is that previous findings of differences among chimpanzee communities may have resulted from statistical artifacts. While Crockford et al. (2004) attempted to control for confounding factors, their sample size of only three individuals per community increases the possibility that apparent differences could emerge by chance. As evidenced from a simulation study (Loken & Gelman, 2017), noisy data with small sample sizes can lead to false positives. For the analyses that were most comparable across this study and that of Crockford et al. (2004) (those focused on climax scream and entire call), our study included slightly more individuals per community (5−7 individuals per community compared to 3 individuals per community in Crockford et al. [2004]). Hence should have detected any differences among communities that were similar in effect size to those reported by Crockford et al. (2004). However, because our sample size remains modest, we could have failed to detect differences if the effect size at Gombe is lower than that at Taï, and hence we cannot rule out the potential for false negatives either. Further, neither our study, nor Crockford et al. (2004) controlled for individual‐level factors such as age, body size, health condition, and rank that could influence the acoustic structure. In addition, no studies have been able to quantitatively control for other factors such as the influence of habitat differences and sound environments that Mitani et al. (1999) suggested could be important. Hence, we argue that firm conclusions regarding chimpanzee vocal learning ability require further study, ideally with a larger number of sampled individuals per community. Furthermore, reanalyses of existing data with different methods such as Bayesian inference is another potential avenue for future research.

Our results reinforce the importance of replicating findings in animal behavior research. A key feature of scientific discovery is seeking results that are consistently reproducible (Burman et al., 2010; Johnson, 2002; Lamal, 1990; Popper, 1959). In recent decades, analyses of studies in several scientific disciplines, including fields as diverse as psychology and medicine, have found that most scientific findings fail to be reproduced by subsequent studies, leading to what has been called the replication crisis (Ioannidis, 2005; Wiggins & Chrisopherson, 2019). One factor contributing to this crisis is that studies replicating existing findings are rarely conducted, and are implicitly discouraged through reviewer bias against them (Neuliep & Crandall, 1993). Given that field studies in animal behavior typically have smaller sample sizes than studies in psychology or medicine, it is likely that the field of animal behavior is in even greater need of replication to test the validity of previous results with sufficient sample sizes (Johnson, 2002). Within animal behavior, the need for replication may be particularly acute for species such as chimpanzees, for which field conditions make it challenging to obtain sample sizes sufficient to be confident in results. Long‐term data from multiple field sites have proven essential for providing sufficient sample sizes for a range of topics (e.g., culture: Whiten et al., 1999; reproductive cessation: Emery Thompson et al., 2007; lethal aggression: Wilson et al., 2014). Such collaboration across long‐term studies will be essential for answering questions about vocal communication as well.

## AUTHOR CONTRIBUTIONS

**Nisarg P. Desai**: conceptualization (lead); data curation (equal); formal analysis (lead); investigation (lead); methodology (lead); project administration (lead); visualization (lead); writing–original draft (lead); writing–review & editing (lead). **Pawel Fedurek**: data curation (equal); writing–review & editing (equal). **Katie E. Slocombe**: data curation (equal); writing–review & editing (equal). **Michael L. Wilson**: conceptualization (equal); data curation (equal); funding acquisition (lead); investigation (equal); methodology (equal); project administration (equal); supervision (equal); writing–original draft (equal); writing–review & editing (equal).

## CONFLICT OF INTEREST

The authors declare no conflict of interest.

## Supporting information

Supplementary information.

## Data Availability

The R code and data for the analyses are available from GitHub at https://github.com/desai-nisarg/Gombe-dialects. Audio recordings from Gombe are available from M. L. W. and from Kanyawara available from P. F. at reasonable request.
