# Supplementary material for: Chimpanzee pant‐hoots encode individual information more reliably than group differences
Source: Am J Primatol. 2022 Sep 12;84(11):e23430. doi: 10.1002/ajp.23430 (PMC9786991; doi:10.1002/ajp.23430)

Figure S1 (a): Differences in the number of letdown components between contexts at individual and community levels.


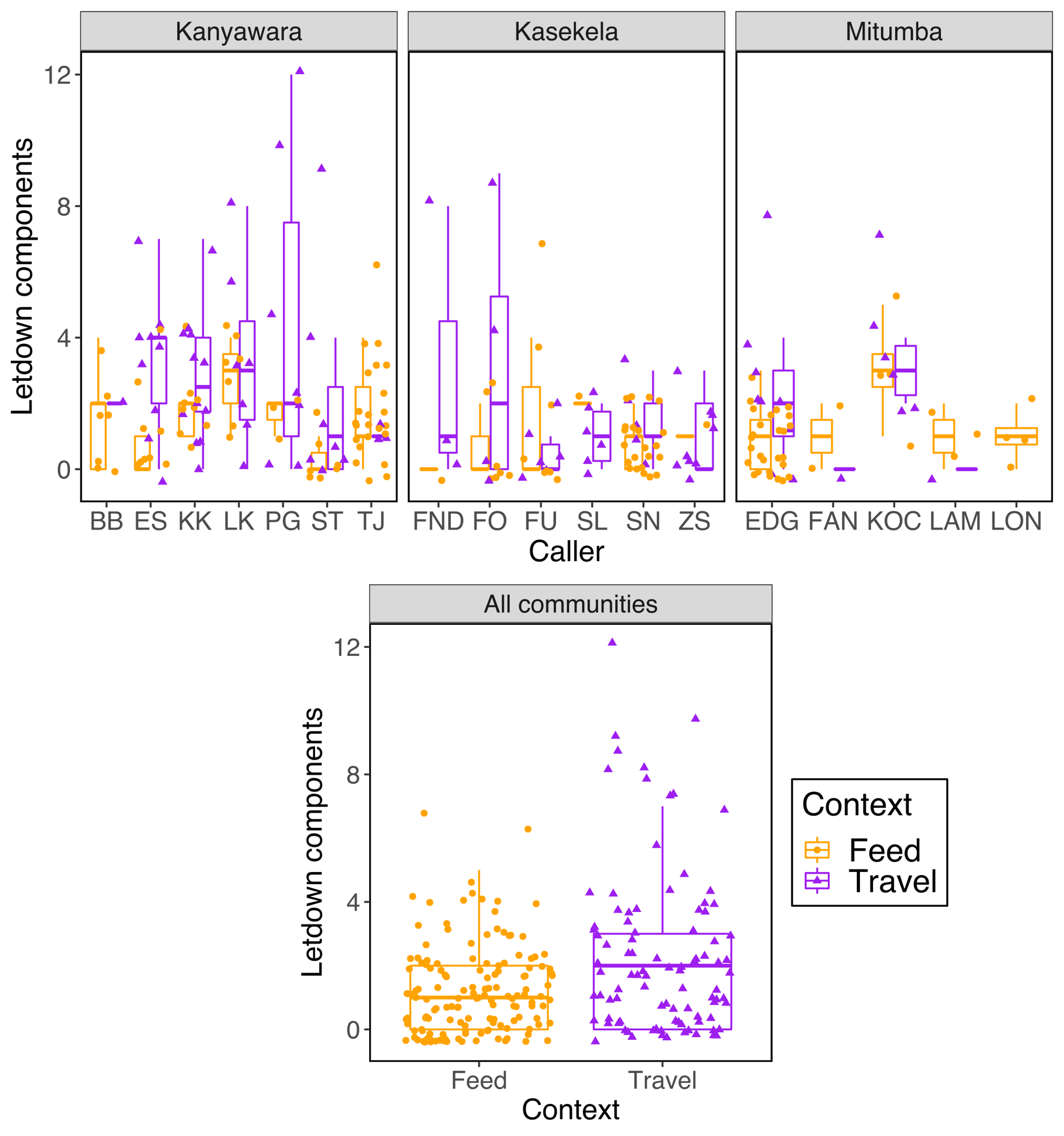


Figure S1 (b): Differences in the number of build-up components between contexts at individual and community levels.


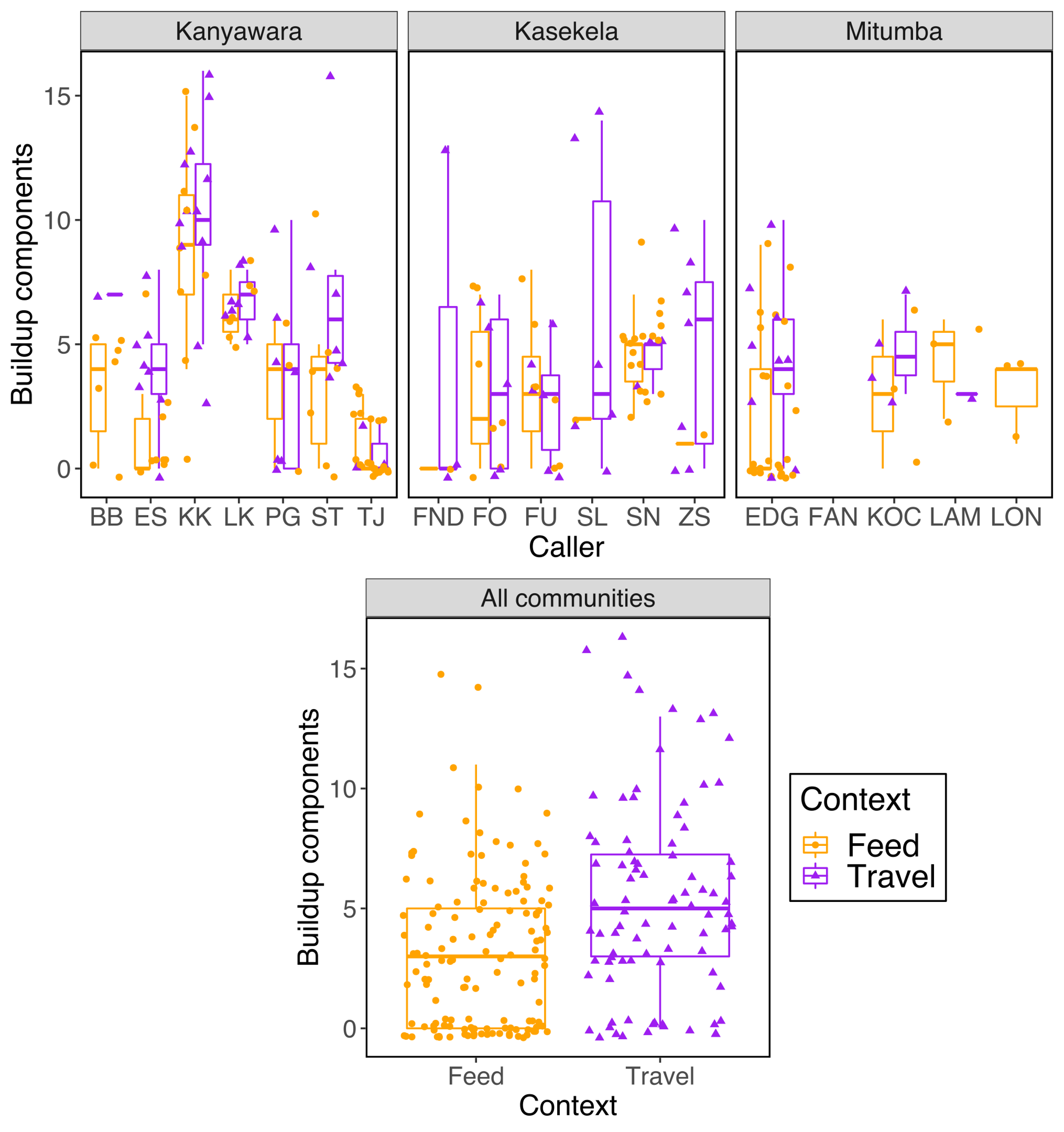


Figure S1 (c): Differences in the number of climax components between contexts at individual and community levels.


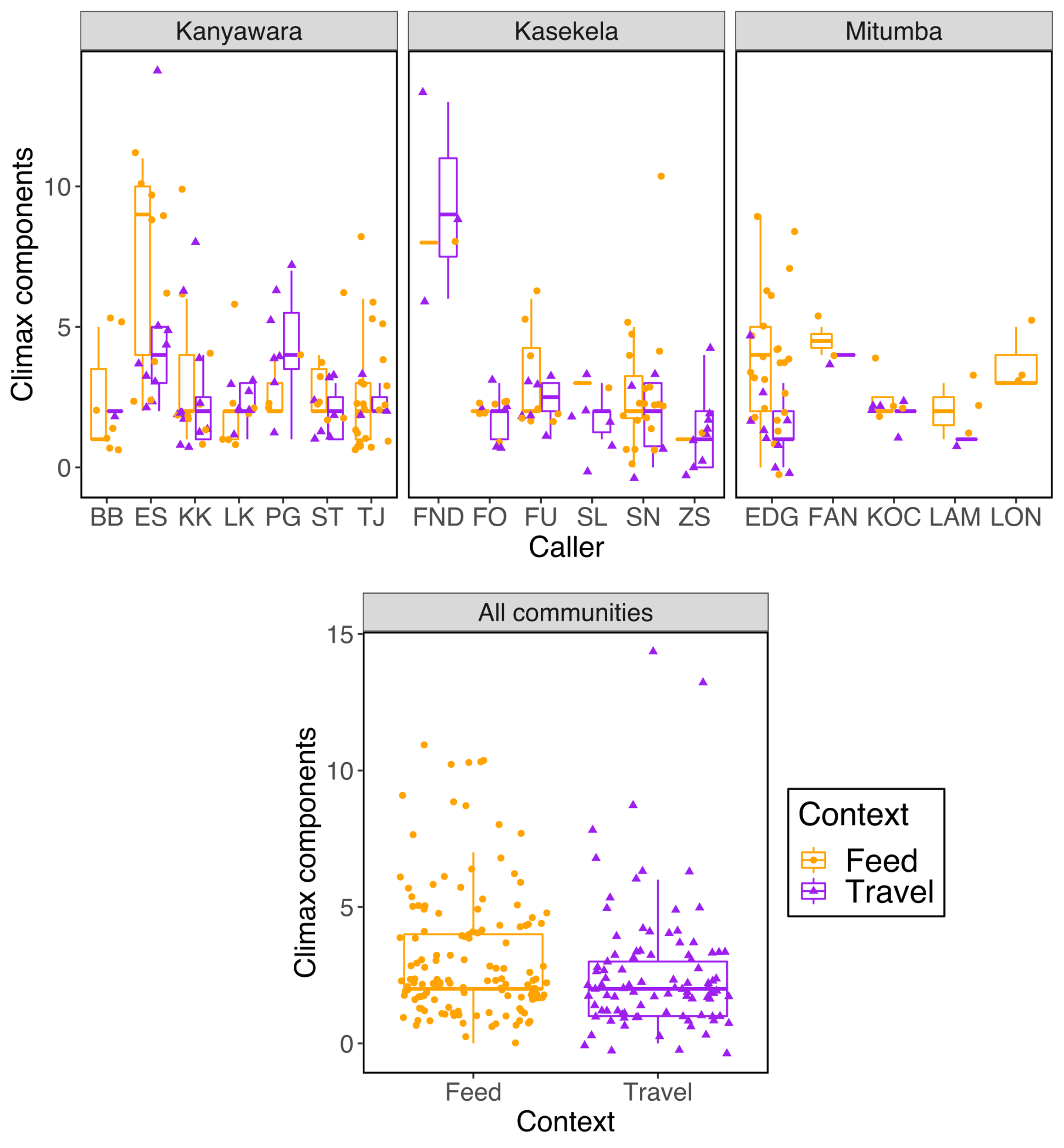


Performing the analyses in Table 6 after removing outliers (points outside 2SD from mean on PC1 or PC2):

i) For climax screams: the 3-community analysis p-value changed from 0.016 (in Table 6) to

0.019. The 2-neighboring community analysis p-value changed from 0.089 to 0.076 (in Table

6).

Figure S2 (a): Climax scream features (Figure 3 (c) in the manuscript) with (left) and without (right) outliers:

ii) For entire calls: the 3-community analysis p-value changed from 0.079 (in Table

6) to 0.089. The 2-neighboring community analysis p-value changed from 0.272 (in Table 6) to

0.215.

Figure S2 (b): Acoustic features from entire calls (Figure 3 (d) in the manuscript) with (left) and without (right) outliers:

Table S1: Mean ± SD of acoustic features found to have group differences in Crockford et al. (2004) ^a^, Mitani et al. (1999) ^b^, Mitani et al. (1992) ^c^:

| Acoustic variable | Context | Community | | |
| --- | --- | --- | --- | --- |
|  |  | Kanyawara | Kasekela | Mitumba |
| Climax: scream duration^a^ | Feed | 781.14 ± 249.02 | 988.11 ± 237.22 | 680.56 ± 189.45 |
|  | Travel | 677.75 ± 192.09 | 864.56 ± 223.24 | 497.89 ± 196.51 |
| Climax: maximum peak frequency^a^ | Feed | 2308.23 ± 1003.87 | 3308.15 ± 2042.71 | 2194.85 ± 1384.31 |
|  | Travel | 2359.54 ± 1548.16 | 3152.52 ± 2008.58 | 1454.53 ± 967.32 |
| Climax: minimum peak frequency^a^ | Feed | 554.02 ± 164.16 | 504.59 ± 200.6 | 545.03 ± 216.51 |
| Climax: mean tonal quality^a^ | Travel | 124.43 ± 47.75 | 91.79 ± 34.52 | 96.41 ± 51.29 |
| Climax: maximum tonal quality^a^ | Feed | 463.48 ± 195.96 | 370.28 ± 133.33 | 308.74 ± 131.3 |
| Climax: minimum F0^a^ | Travel | 656 ± 131.35 | 637.28 ± 179.35 | 594.12 ± 116.94 |
| Climax: maximum F0^b^ | Feed | 1328.43 ± 345.35 | 1180.21 ± 235.17 | 1082.72 ± 303.94 |
|  | Travel | 1293.59 ± 302.2 | 1256.03 ± 363.49 | 1091.29 ± 270.06 |
| Climax: mean F0^b,c^ | Feed | 1108.35 ± 267.16 | 970.74 ± 199.7 | 884.69 ± 259.74 |
|  | Travel | 1067.63 ± 272.65 | 998.24 ± 279.03 | 864.47 ± 207.09 |
| Climax: frequency range of F0^c^ | Feed | 649.2 ± 256.29 | 561.18 ± 198.22 | 490.82 ± 193.71 |
|  | Travel | 637.59 ± 209.71 | 618.76 ± 258.78 | 497.18 ± 234.3 |
| Buildup: phase duration^b^ | Feed | 1.38 ± 1.48 | 2.12 ± 1.39 | 1.3 ± 1.57 |
|  | Travel | 2.57 ± 1.69 | 2.07 ± 2.02 | 2.18 ± 1.35 |
| Build-up: element duration^b,c^ | Feed | 301.06 ± 67.02 | 361.86 ± 103.44 | 342.6 ± 89.1 |
|  | Travel | 280.85 ± 59.1 | 340.85 ± 71.58 | 345.65 ± 87.72 |
| Build-up: rate^b,c^ | Feed | 1.35 ± 1.21 | 1.73 ± 0.91 | 1.06 ± 1.12 |
|  | Travel | 2.19 ± 0.99 | 1.49 ± 1.09 | 1.75 ± 0.81 |
| Build-up: rate of first half^a^ | Feed | 1.24 ± 1.15 | 1.49 ± 0.77 | 1.03 ± 1.11 |
| Build-up: acceleration rate^a^ | Travel | -0.02 ± 0.4 | 0.1 ± 0.38 | 0.08 ± 0.2 |
| Build-up: number of exhalation elements^a^ | Travel | 6.36 ± 4.28 | 4.2 ± 4.17 | 4.36 ± 2.68 |

Figure S3 (a): Buildup to letdown duration at individual and community levels.


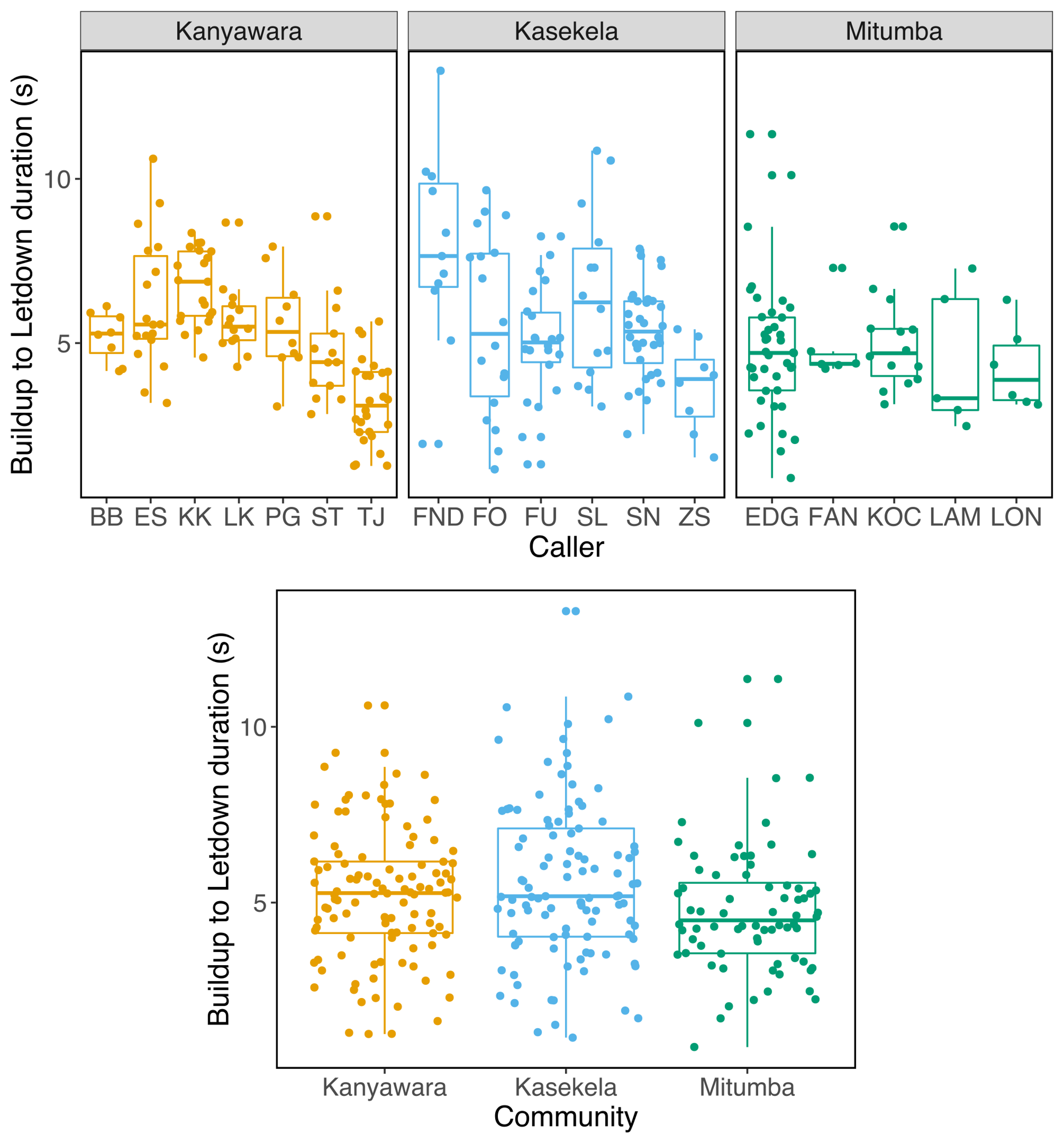


Figure S3 (b): Proportion of calls with build-up at individual and community levels.


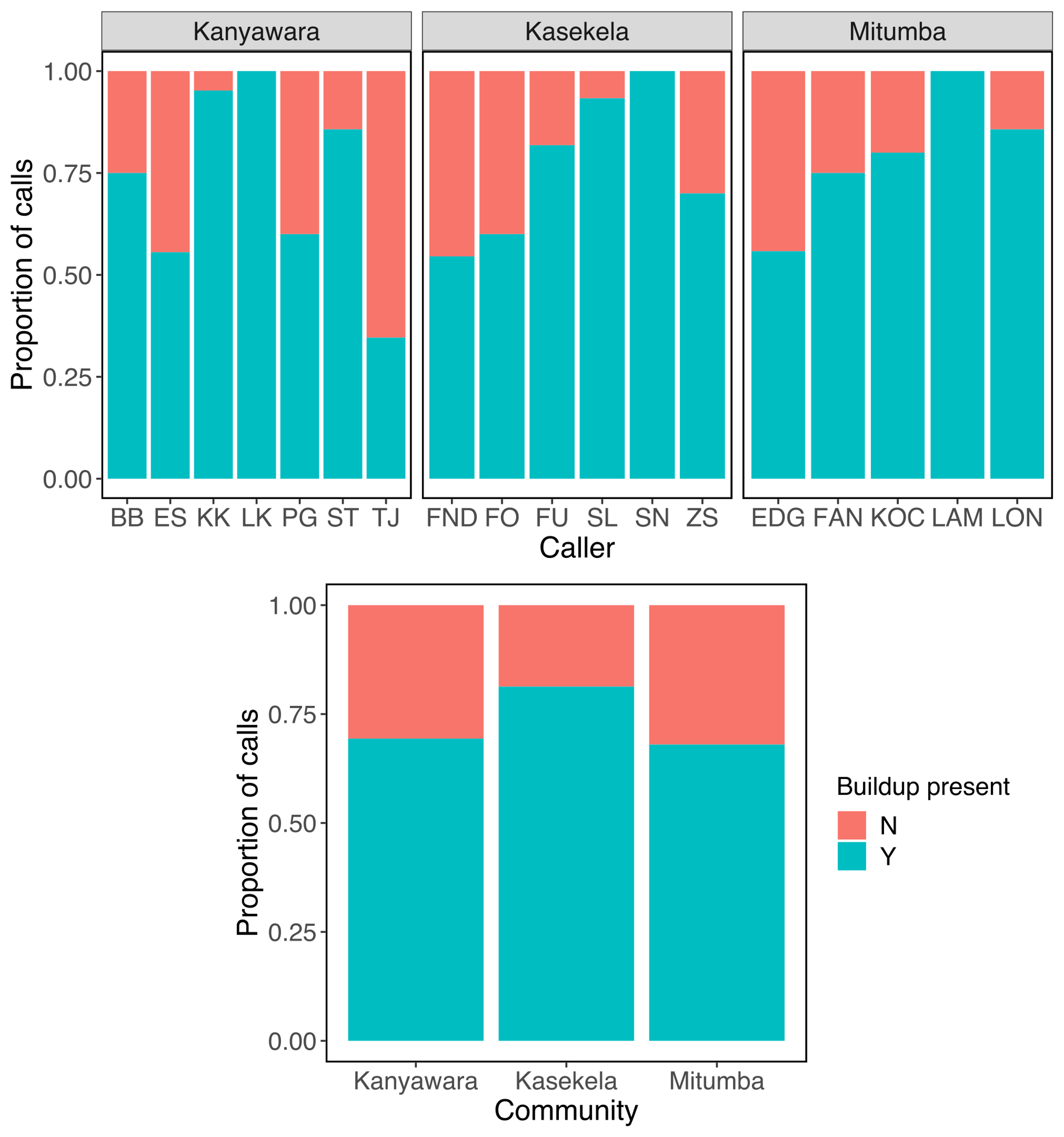


Figure S2 (c): Build-up duration at individual and community levels.


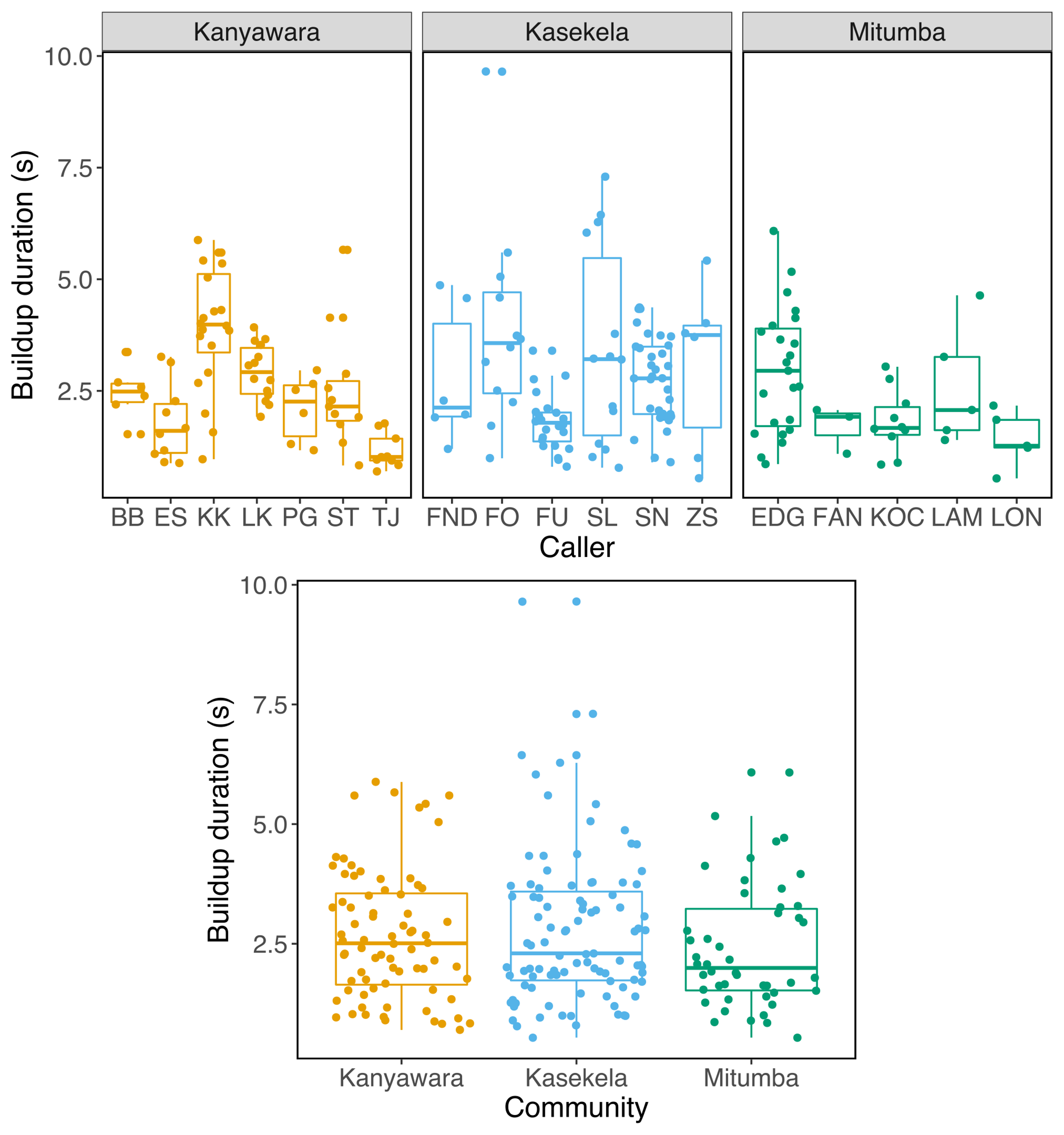


Figure S3 (d): Number of build-up exhalation elements at individual and community levels.


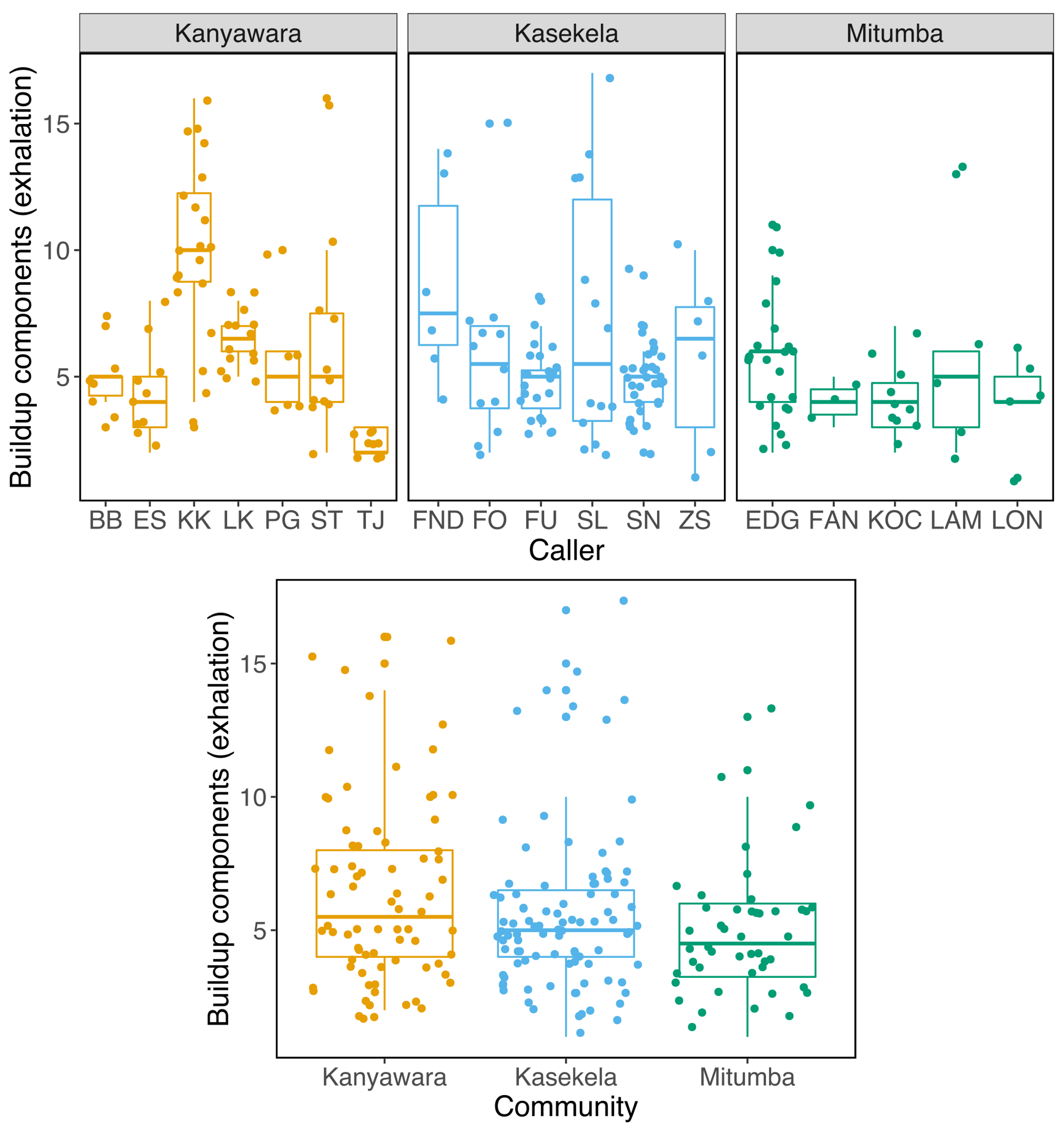


Figure S3 (e): Rate of build-up at individual and community levels.


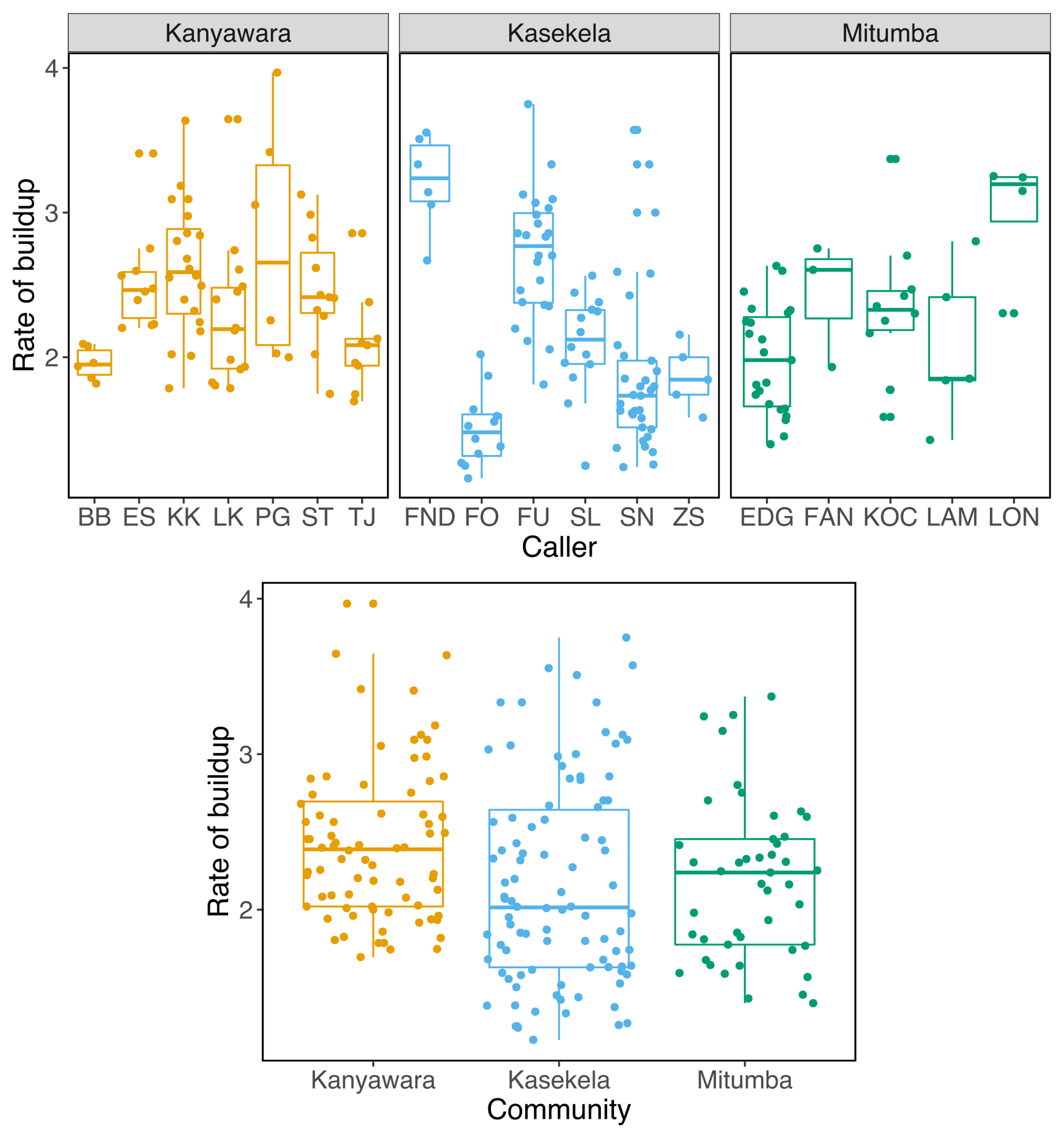


Figure S3 (f): Climax duration at individual and community levels.


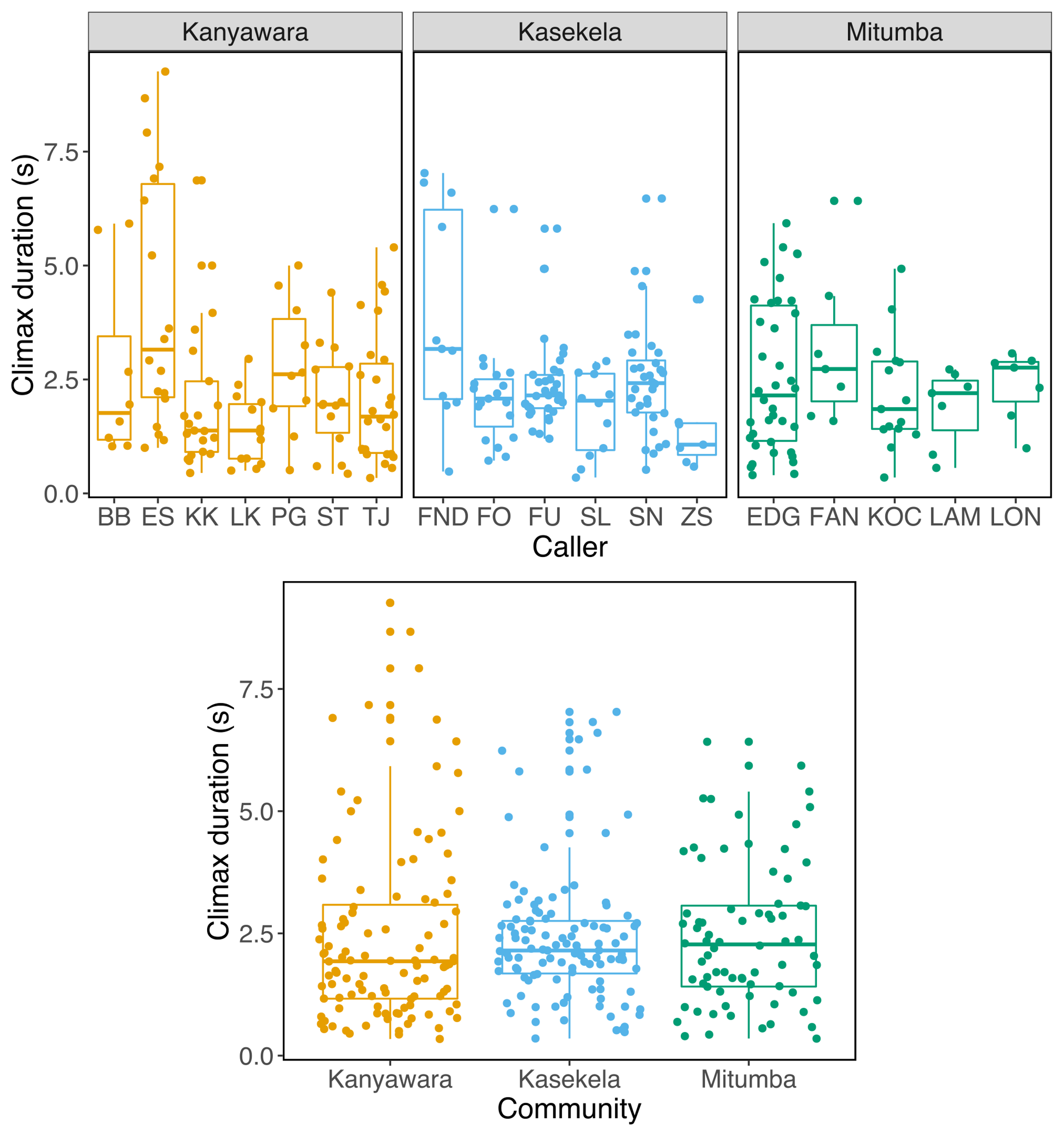


Figure S3 (g): Number of climax components at individual and community levels.


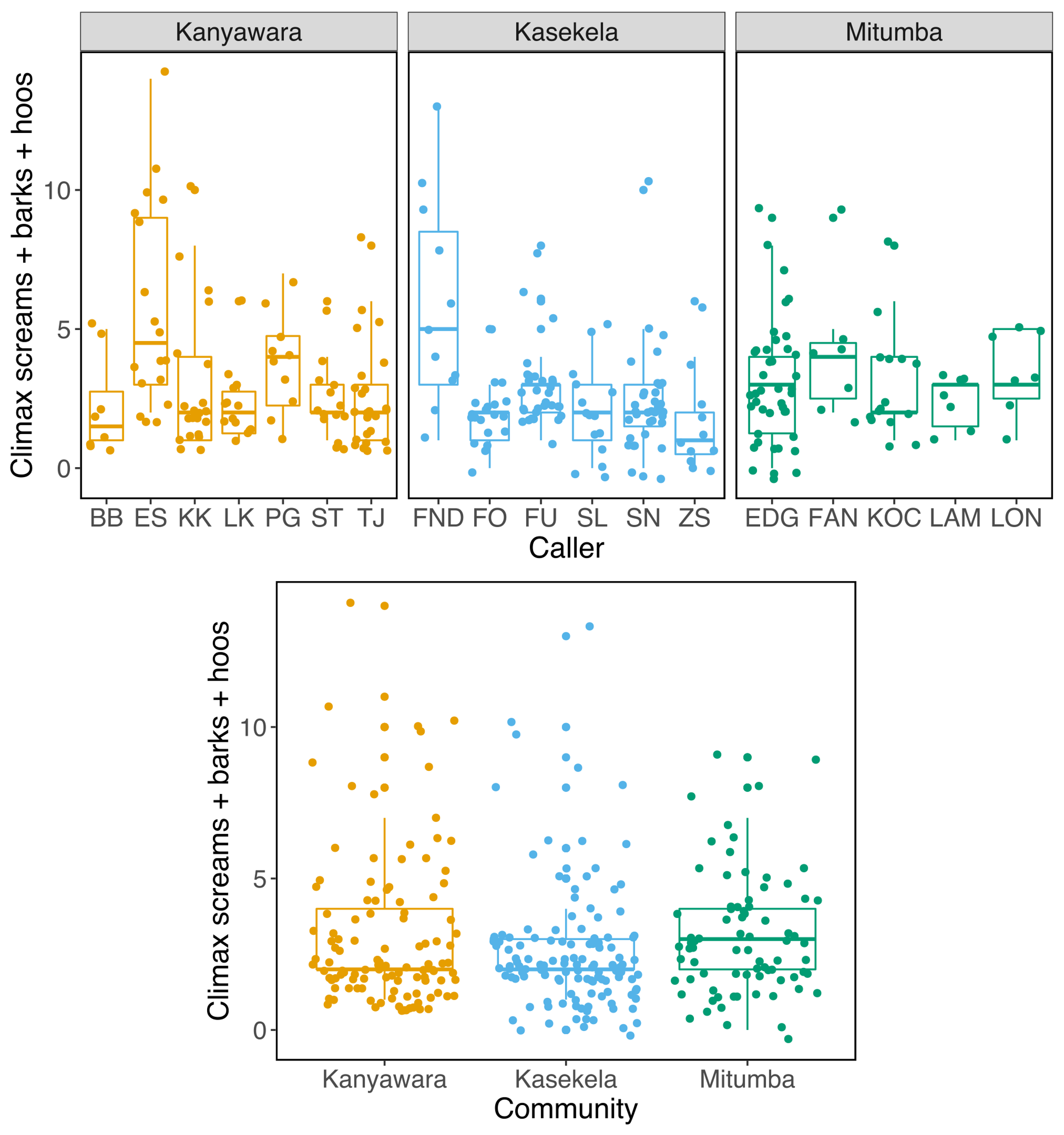


Figure S3 (h): Number of climax screams at individual and community levels.


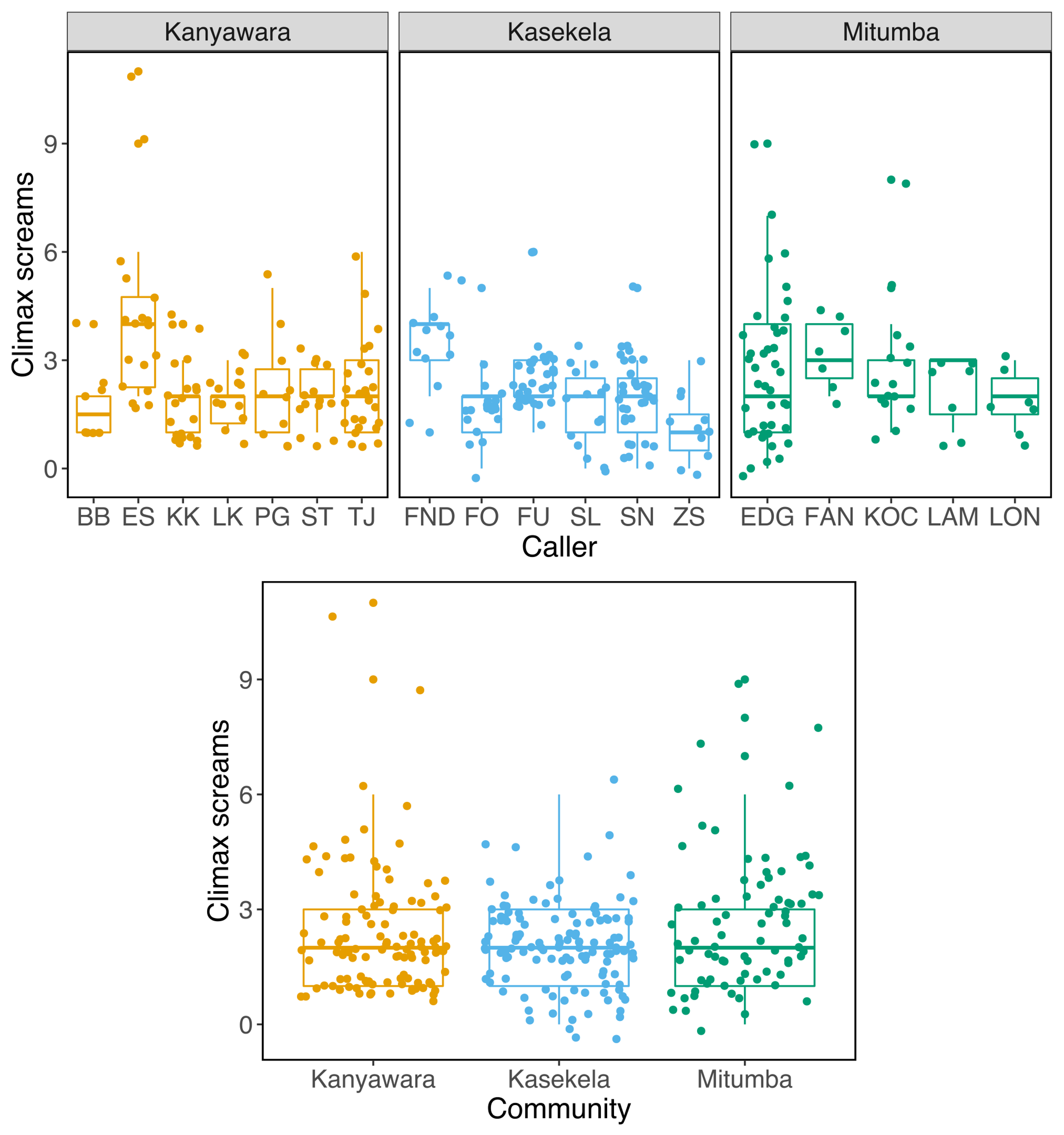


Figure S3 (i): Proportion of climax components that are screams at individual and community levels.


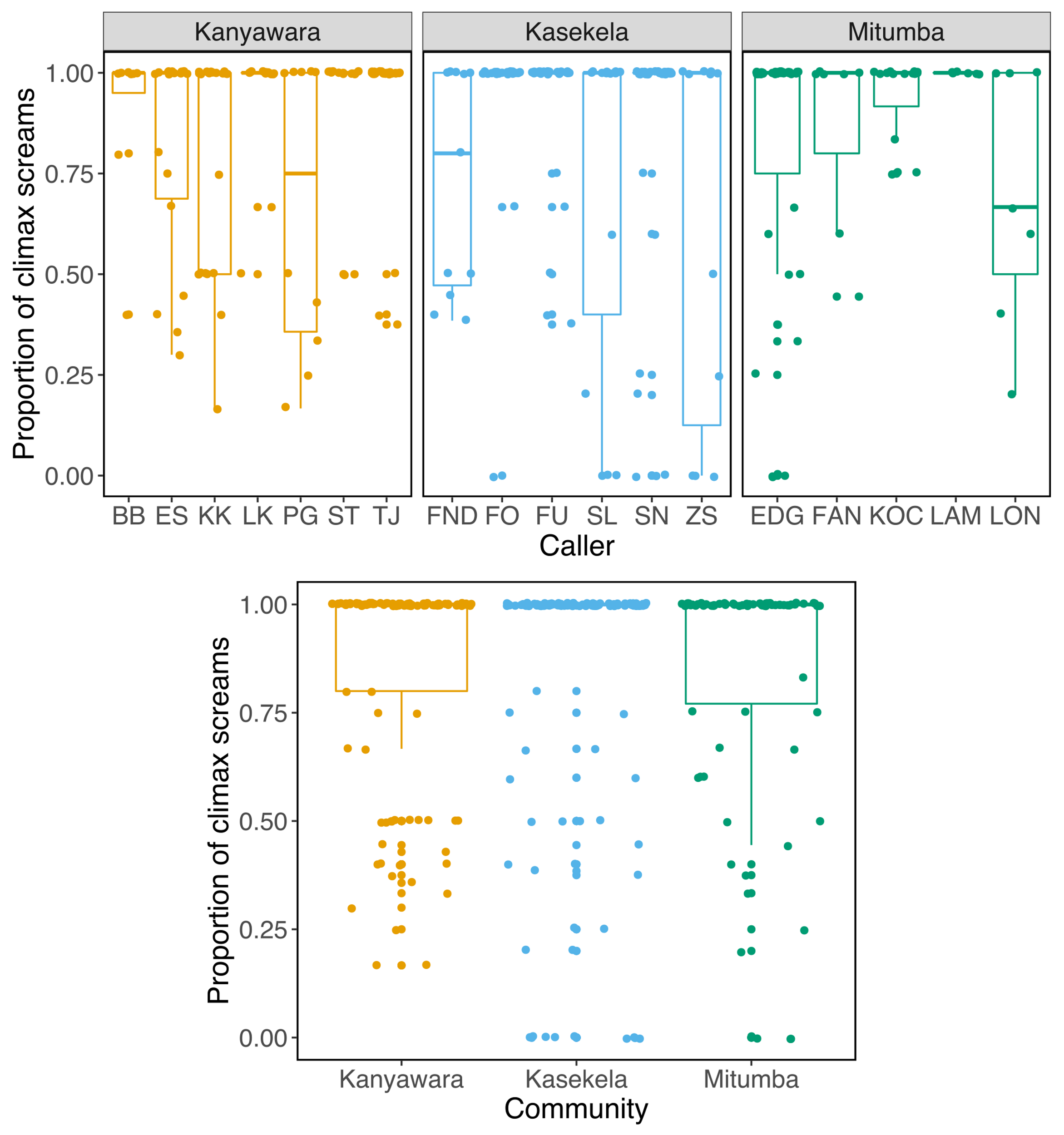


Figure S3 (j): Proportion of calls with letdown present at individual and community levels.


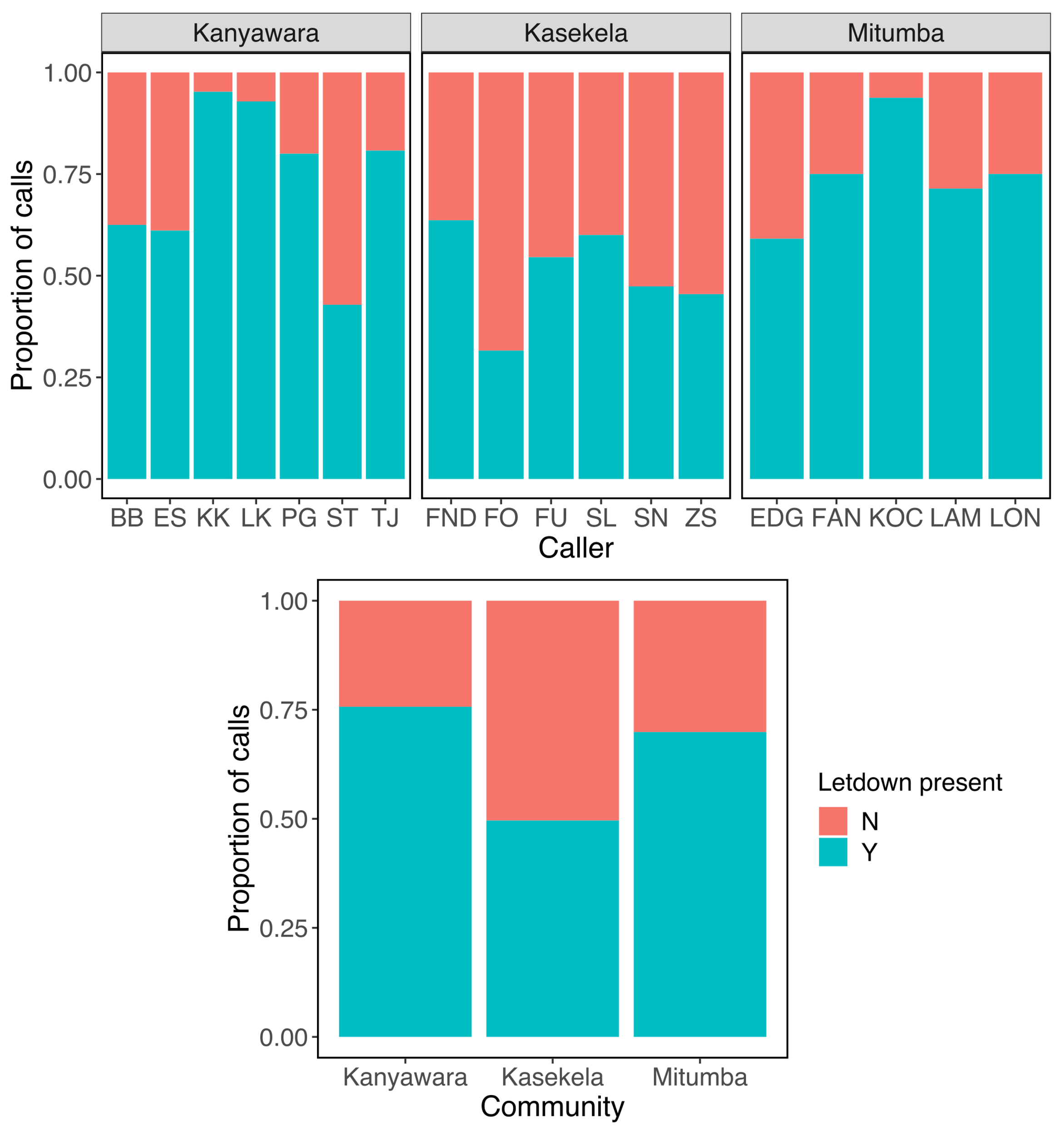


Figure S3 (k): Number of letdown components at individual and community levels.


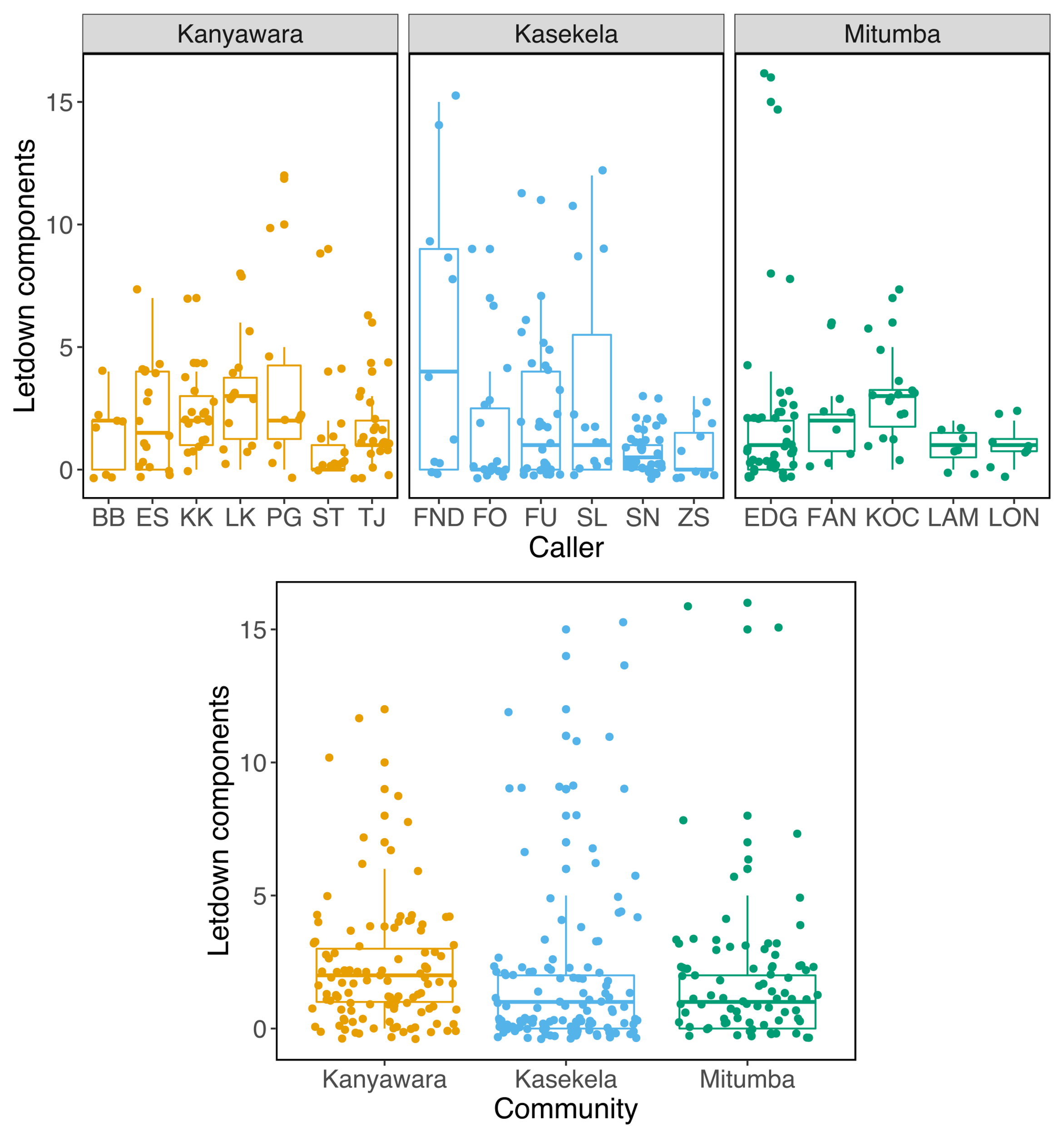


Figure S3 (l): Proportion of calls with drumming present at individual and community levels.


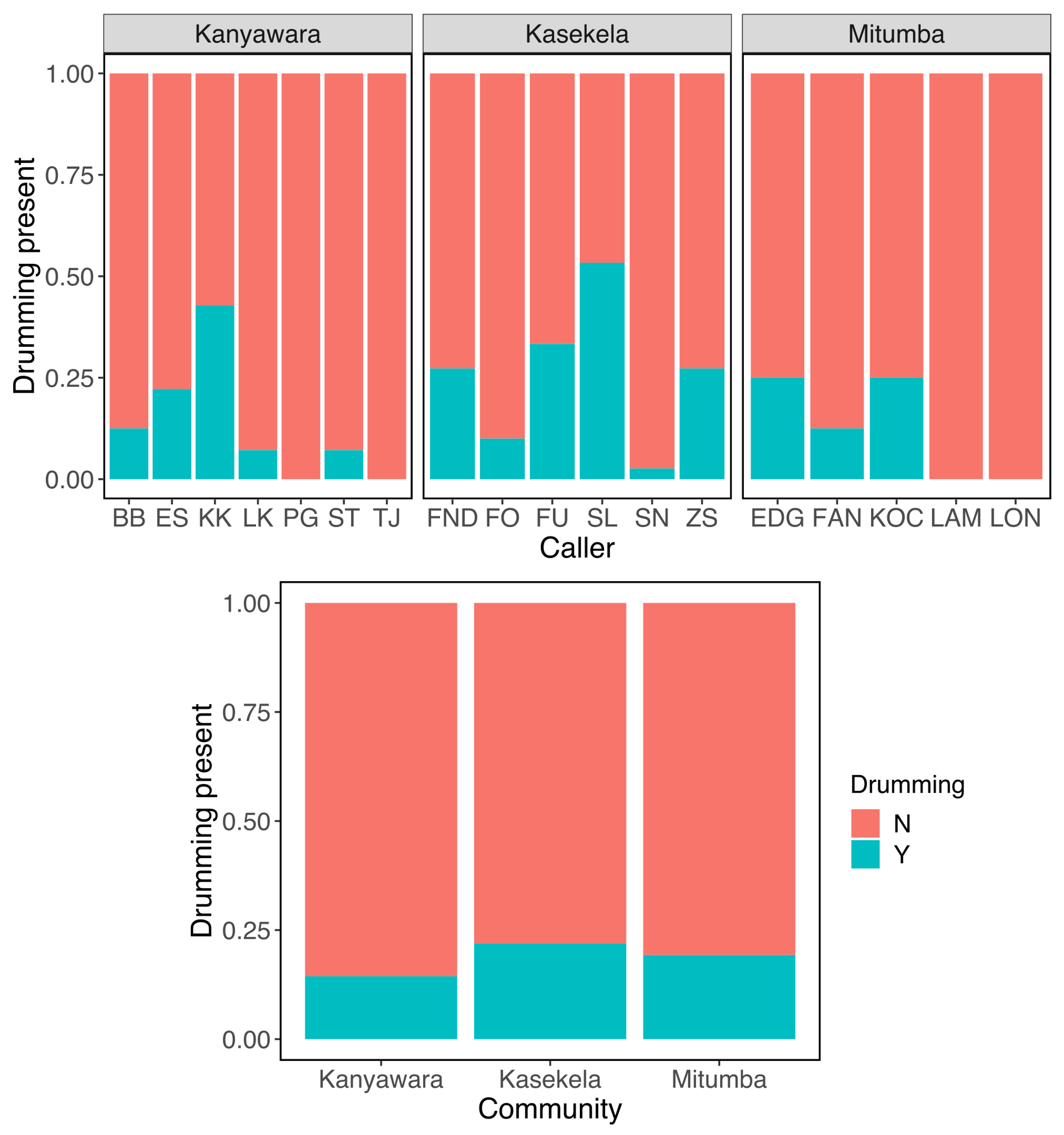


Figure S3 (m): Number of drum beats at individual and community levels.


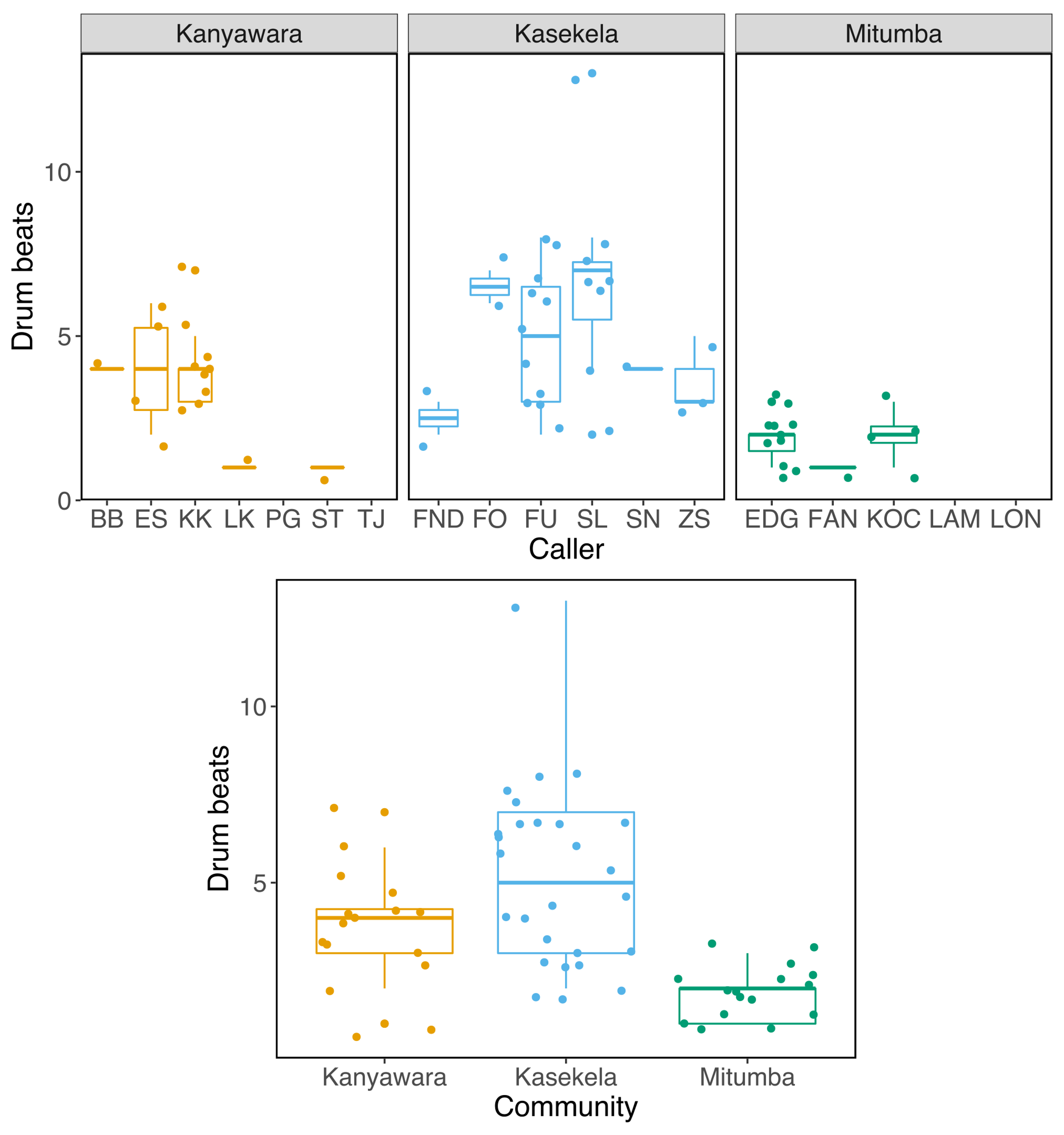

Supplement: Supplementary file 1 — Supplementary information. [file AJP-84-e23430-s001.docx]
